# Microwave-Assisted Extraction of Hemp Seed Oil: Process Optimization for Enhancing Oil Yield and Bioactive Compound Extractability

**DOI:** 10.1155/ijfo/7381308

**Published:** 2025-04-24

**Authors:** Aymane Allay, Chaymae Benkirane, Abdessamad Ben Moumen, Youssef Rbah, Marie-Laure Fauconnier, Hana Serghini Caid, Ahmed Elamrani, Farid Mansouri

**Affiliations:** ^1^Laboratory of Agricultural Production Improvement, Biotechnology, and Environment, Faculty of Sciences, Mohammed I University, Oujda, Morocco; ^2^Laboratory of Chemistry of Natural Molecules, Gembloux Agro-Bio Tech, University of Liège, Gembloux, Belgium; ^3^Higher School of Education and Training, Mohammed I University, Oujda, Morocco

**Keywords:** hemp seed oil, microwave-assisted extraction, oxidative stability, phenolic compounds, tocopherols

## Abstract

Hemp seed oil is a valuable source of unsaturated fatty acids. However, its high degree of unsaturation makes it highly susceptible to oxidation, which can compromise its quality and nutritional value. Enhancing its stability can be achieved by incorporating antioxidant compounds naturally present in the seeds. A deeper understanding of the interactions between the extraction process and the oil's composition would provide valuable insights for optimizing both its stability and bioactive properties. In this context, this study was aimed at investigating and optimizing the microwave-assisted extraction of hemp seed oil, enriched with bioactive compounds, using response surface methodology. Three extraction factors were investigated: microwave power (600, 750, and 900 W), cosolvent percentage (0%, 5%, and 10% ethanol relative to hexane), and extraction time (5, 10, and 15 min). Several responses were studied, including oil yield, total phenolic content (TPC), total tocopherols, oxidative stability index (OSI), chlorophylls, carotenoids, quality indices (peroxide value and conjugates [diene and triene]), and color (*L*∗, *a*∗, *b*∗, *C*∗⁣_ab_, and *h*_ab_). The optimum extraction conditions were 800 W, 7.5% EtOH, and 13.60 min, reaching a maximum yield of 30.69%. The resulting oil showed a high OSI (28.60 h) and a richness in TPC, tocopherols, carotenoids, and chlorophylls (88.55, 510.64, 27.21, and 99.68 mg/kg oil, respectively) along with an acceptable oxidation quality and intense color parameters (*L*∗ = 33.54, *a*∗ = −4.01, and *b*∗ = 3.17). Furthermore, a detailed analysis of phenolic compounds using HPLC-DAD/ESI-MS was carried out on microwave-extracted oils. The finding showed that both variables (power and %EtOH) resulted in notable fluctuations in the phenolic profile of the extracted oil. The contents of phenolic acids (13.48 mg/kg), hydrocinnamic acid amides (9.97 mg CTE/kg), and lignanamides (16.18 mg CTE/kg) identified in the hemp seed oil were highest under 600 W, 10% ethanol, and 10 min.

## 1. Introduction

Hemp seed oil is the prevalent edible derivative of hemp (*Cannabis sativa* L.). It has a pleasant nutty flavor and slightly bitter aftertaste. *Cannabis* seed oil has proven cardiovascular health benefits by lowering cholesterol levels and hypertension [1]. It could also be used as a dietary supplement due to its polyunsaturated fatty acid profile. *Cannabis* seed oil improves skin quality through its moisturizing properties, reducing the need for topical medication for dryness. Besides, *Cannabis* seed oil has potential uses in the cosmetics industry as a sunscreen due to its ability to absorb UV rays and its high tocopherol content [2, 3]. The oil belongs to the category of unsaturated oils, containing up to 90% unsaturated fatty acids, of which 70% to over 80% are polyunsaturated fatty acids. While unsaturated oils are of undoubted nutritional interest, they are also highly sensitive to oxidation reactions, which accelerate the rancidity of the oil.

Several bioactive compounds, such as phenolic compounds, are essential for protecting the oil against oxidation and extending its shelf life. These antioxidants found in the oil are naturally present in the seeds. The phenolic compounds in hemp seeds belong mainly to the phenylpropionamide class, which includes phenylamides and lignanamides. In particular, hemp seeds are rich in molecules such as caffeoyltyramine, cannabisin A, and cannabisin B [4–6]. Various phenylpropionamides isolated from different parts of hemp seeds (whole seeds, hulls, or flours) have demonstrated interesting biological properties [2]. However, other phenolic compounds have been identified in hemp seed oil, mainly phenolic acids, such as 4-hydroxybenzoic, benzoic, caffeic, p-coumaric, and sinapic acids [7–9]. Although phenolic compounds are abundant in hemp seeds, their passage into the oil is limited due to their high polarities, making the oil very sensitive to oxidation. Knowledge of the interactions between the extraction process and the composition of the oil would enable us to identify the possibility of improving oil quality. Improved oil quality could be achieved by entraining antioxidant compounds from the seeds. In addition, processes need to be adapted to produce oils containing compounds of interest.

Conventional extraction methods for hemp seed oil include mechanical pressing and solvent-based techniques. Mechanical extraction, using screw or hydraulic presses, is widely favored for producing cold-pressed oils with minimal processing, as it preserves natural nutrients and is environmentally friendly [10, 11]. However, this method often yields lower oil recovery compared to solvent extraction. The latter, typically employing hexane, has been criticized for its toxicity and environmental impact, leading to the exploration of greener alternatives such as supercritical CO_2_. Supercritical fluid extraction is a green technology, enjoying environmental and operational advantages over conventional methods. Supercritical CO_2_, thanks to its gas-like diffusion properties and liquid-like solvation power, makes extraction efficient even at relatively low temperatures. As a result, many researchers have adopted this method for hemp seed oil extraction [12, 13].

Microwave-assisted extraction (MAE) is an emerging technique increasingly used for its ability to reduce extraction time, decrease solvent consumption, and improve efficiency compared to conventional methods. This technique harnesses microwave energy to rapidly heat plant materials, optimizing the extraction of oil and bioactive compounds. Its effectiveness has been demonstrated across various oilseeds. Zhong et al. [14] reported a 94.21% oil recovery from *Moringa oleifera* seeds using MAE, surpassing Soxhlet extraction. Similarly, Ibrahim et al. [15] highlighted the solvent-dependent efficiency of MAE, with ethyl acetate yielding 72.75% oil from sandbox seeds compared to 56.25% for n-hexane, underscoring the importance of solvent polarity in bioactive compound retention.

For hemp seed oil, recent studies have validated MAE's potential, demonstrating shorter extraction times compared to conventional methods [16, 17]. While these studies provide valuable insights into MAE-derived hemp seed oil, none has specifically investigated the effects of key extraction parameters (microwave power, cosolvent ratio, and extraction time) on the extractability of bioactive compounds, particularly phenolic compounds, and their role in improving the oxidative stability of the extracted oil.

Addressing these limitations, this study pioneers a comprehensive optimization of MAE for hemp seed oil, focusing on dual objectives: maximizing yield and enhancing bioactive compound extractability. Notably, our work introduces high-performance liquid chromatography (HPLC)–diode array detector (DAD)/electrospray ionization source (ESI)–MS analysis for the precise identification and quantification of phenolic compounds, representing a significant advancement over previous methodologies. By elucidating the synergies between extraction parameters and bioactive compound retention, this research provides actionable insights for producing stable, nutrient-rich hemp seed oil, aligning with both industrial and environmental priorities.

We hypothesized in this work that (i) MAE using ethanol as a cosolvent may affect the extraction efficiency of bioactive components (phenolic compounds, tocopherols, and pigments) of hemp seed oil and that (ii) hemp seed oil extracted with MAE contains higher amounts of bioactive compounds with a high oxidative stability index (OSI). The study of these attributes is of extreme importance in the pharmaceutical and cosmetic industries since hemp seed oil is known for its protective and curative roles in human health. For this purpose, this study comprehensively explored MAE of hemp seed oil by investigating the impact of power (600, 750, and 900 W), time (5, 10, and 15 min), and cosolvent amount (0%, 5%, and 10% of ethanol relative to hexane) on oil yield and quality using response surface methodology (RSM). This study specifically evaluated changes in yield, phenolic composition, tocopherols, pigments (chlorophylls and carotenoids), oxidative stability, color, peroxide value (PV), and conjugate (diene and triene) characteristics.

## 2. Materials and Methods

### 2.1. Plant Material

Seeds of a local ecotype of hemp (*Cannabis sativa* L.), known as Beldia, were kindly provided by the National Agency of Medicinal and Aromatic Plants (ANPMA) in Taounate, Morocco. *Cannabis* plants were cultivated at the ANPMA experimental station located in northern Morocco. The seeds were collected at full maturity in July, aligning with the common practice of Moroccan hemp growers. After cleaning the seeds from impurities, their moisture content was determined by drying at 100°C ± 3°C in accordance with the AOAC standard 925.40 [18]. The moisture content was 5.13% ± 0.11%. Subsequently, the samples were ground with a laboratory grinder and sifted using a 500-*μ*m mesh sieve to obtain a homogenous particle size.

### 2.2. Experimental Design

RSM is a statistical approach utilizing data from a suitable experimental design to optimize processes and analyze complex relationships between input variables and responses. In this context, the RSM method, employing the Box–Behnken design (BBD), was conducted to ascertain the optimal conditions for MAE of hemp seed oil. The selection of experimental variables, that significantly influenced microwave oil extraction, was based on preliminary experiments. The outcomes guided the selection of three variables due to their substantial impact on oil yield: microwave power, cosolvent percentage (percent ethanol relative to hexane), and extraction time. A total of 17 experiments were generated based on the three-factor BBD, incorporating five replications at the central point (Table 1). The chosen response parameters encompassed oil yield, total phenolic content (TPC), total tocopherols, OSI, chlorophylls, carotenoids, quality indices (PV and conjugates [diene and triene]), and oil color (*L*∗, *a*∗, *b*∗, *C*∗⁣_ab_, and *h*_ab_).

The relationships between the studied responses and the three extraction variables were modeled using a second-order polynomial equation (1):

(1) $$\begin{array}{c} Y=\beta_{0}+\overset{k}{\underset{i=1}{\sum }}\beta_{i}X_{i}+\overset{k}{\underset{i=1}{\sum }}\beta_{ii}X_{i}^{2}+\overset{k}{\underset{1\leq i\leq j}{\sum }}\beta_{ij}X_{i}X_{j} \end{array}$$

where *Y* is the studied response; *X*_*i*_ and *X*_*j*_ are the independent variables; *β*_0_ stands for the intercept; and *β*_*i*_, *β*_*ii*_, and *β*_*ij*_ represent the regression coefficients for the linear, quadratic, and interaction effect terms.

### 2.3. MAE of Hemp Seed Oil

A Samsung microwave (1.9 cu. ft. Smart Over-the-Range Microwave) was adapted for MAE and employed in this study. The dimensions of the microwave PTFE-lined cavity were: 29.9375^″^ *W* × 16^″^ *D* × 17.125^″^ *H*. Each experiment involved weighing 30 g of crushed and sieved hemp seeds, followed by the addition of pure n-hexane or a mixture of n-hexane and ethanol with varying ethanol percentages (0%–10%), maintaining a constant solid-to-liquid ratio of 1:10 (selected based on preliminary study results). The seed–solvent mixture was put in a flask and introduced into the microwave oven equipped with a cooler to condense the solvent evaporated by radiation. The extraction process's power and duration were adjusted according to the experimental design. All extractions were carried out in triplicate. After each experiment, the extraction solvent was separated from the residual solids using 1 mm filter paper. The remaining cake were washed three times with the solvent used for extraction. The collected solvent was centrifuged (15 min at 5000 g) and then concentrated using a rotary evaporator (40°C ± 1°C). The obtained oil was stored in dark vials under nitrogen at −20°C ± 2°C for 3 weeks until analysis. All chemical analyses were carried out in triplicate for each oil sample. The yield of the microwave-extracted oil from the 17 experiments was calculated using Equation (2).

(2) $$\begin{array}{c} \text{Oil yield}\left(\%\right)=\frac{\text{the weight of obtained oil}}{\text{the weight of seed powder}}\times 100. \end{array}$$

### 2.4. Analytical Methods

#### 2.4.1. Tocopherol Analysis

The determination of tocopherol content was carried out according to the protocol described by Ben Moumen et al. [19] using HPLC-DAD on a Shimadzu LC-6AD system. The system comprised an Uptisphere 120A° NH_2_ column (150 × 3 mm, 3 *μ*m) coupled to a photodiode array detector. A solution of hexane/2-propanol (99/1, *V*/*V*) was used as mobile phase with a 1 mL/min flow rate. Three wavelengths (292, 296, and 298 nm) were used for tocopherol analysis. Identification and quantification were carried out using commercial tocopherol standards (alpha-, beta-, gamma-, and delta-tocopherols) purchased from Sigma-Aldrich (St. Louis, MO, United States). Tocopherol concentrations were calculated from an external calibration curve using different concentrations of a mixture of tocopherols.

#### 2.4.2. Colorimetric Determination of TPC

To prepare the phenolic extracts, the method described by Ben Moumen et al. [20] was used. A 2.5 g of MAE oil was mixed with 2.5 mL of a methanol/water solution (80/20, *v*/*v*) in a 15 mL tube. After shaking for 5 min, the tubes were centrifuged for 15 min at 5000 g.

The extracts' TPC was quantified using the Folin–Ciocalteu method. For this, 0.1 mL of the methanolic extract was combined with 1 mL of 10-fold diluted Folin–Ciocalteu reagent and 1 mL of a 10% (*w*/*v*) aqueous Na_2_CO_3_ solution. After shaking, the solution was kept in the dark for 90 min, and the absorbance was measured at 760 nm using a UV-visible spectrophotometer. Based on a calibration curve established by gallic acid standard (Sigma-Aldrich, St. Louis, MO, United States) with concentrations ranging from 3.12 to 125 *μ*g/mL, the TPC was expressed in milligram of gallic acid equivalent per kilogram of oil.

#### 2.4.3. Analysis of Phenolic Compounds by HPLC-DAD/ESI-MS^2^

The phenolic compounds were separated using a C18 column (particle size: 3.5 *μ*m, length × internal diameter: 150 × 4.6 mm) on an Agilent 1260 Infinity II HPLC system (Agilent Technologies, United States) equipped with a DAD. The injection volume was 10 *μ*L, and the flow rate was 0.6 mL/min. The elution followed a gradient mode as per the method outlined by Benkirane et al. [4]. The detection of phenolic compounds occurred at wavelengths of 254, 280, 300, and 340 nm [21]. The UV-visible spectra for each compound were recorded between 190 and 600 nm. Chromatographic data were visualized and analyzed using Agilent OpenLAB CDS software. The separated peaks were collected individually at the outlet of the HPLC system and analyzed in negative and positive mode mass spectrometry (Esquire HCT mass spectrometer, Bruker Daltonics, Germany). ESI was used for smart mode ionization with a sputtering voltage of 4500 V, a dry gas temperature of 200°C, a nebulizer of 10 psi, and a dry gas flow rate of 4 L/min. Each precursor ion, after its isolation in the ion trap, was exposed to a collision energy ranging from 1% to 10% and expelled according to *m*/*z* ratio. Data processing was carried out using ACD/labs 2021.2.1 software. Phenolic compounds were identified by comparing their molecular and fragment ion masses and UV spectra with the literature [4–6, 21, 22] and mass databases (MassBank of North America (MoNA)). Quantification was achieved by determining peak areas from the HPLC-DAD profile at 280 nm using an external standard curve (2.6–84 *μ*g/mL) of *N-trans*-caffeoyltyramine (Sigma-Aldrich, St. Louis, MO, United States). The outcomes are presented as milligram *N-trans*-caffeoyltyramine equivalent per kilogram oil due to the limited availability of most phenolic compounds identified in the hemp seed oil in this study. Only hydroxybenzoic acid (0.6–40 *μ*g/mL), benzoic acid (0.6–40 *μ*g/mL), *p*-coumaric acid (0.6–40 *μ*g/mL), and sinapic acid (0.6–40 *μ*g/mL) were quantified using their respective commercial standards purchased from Sigma-Aldrich (St. Louis, MO, United States).

#### 2.4.4. Chlorophyll and Carotenoid Contents

Determination of chlorophyll and carotenoid content (expressed as milligrams per kilogram) in oil samples from the 17 experiments followed the procedure described by Aachary et al. [23]. Briefly, 0.1 g of oil was dissolved in 5 mL of absolute diethyl ether. The resulting solution was vortexed, subjected to ultrasonication for 1 min, and then centrifuged (5000 g, 10 min). Finally, the absorbance was measured at 470, 640, and 663 nm using a spectrophotometer. The content of pigments in the oil was calculated in micrograms of pigments per gram of oil (milligrams per kilogram) using Equations (3) and (4):

(3) $$\begin{array}{c} \text{Chlorophylls a}+\text{b }\left(\text{mg}/\text{kg}\right)=\frac{7.12\times A_{663}+16.80\times A_{640}}{W} \end{array}$$

(4) $$\begin{array}{c} \text{Total Carotenes }\left(\text{mg}/\text{kg}\right)=\frac{\left(1000\times A_{470}\right)-\left(0.52\times \text{Chly a}\right)-\left(7.25\times \text{Chly b}\right)}{226\times W} \end{array}$$

where *A* is the absorbance at wavelengths (663, 640, and 470 nm) and *W* is the weight of hemp seed oil.

#### 2.4.5. Color Analysis

CIELAB parameters (*L*∗, *a*∗, and *b*∗) for oils extracted from the 17 experiments were directly determined using a chromameter (Konica Minolta Chroma Meter CR-410, Measurement area ⌀ 50 mm) equipped with a silicon photocell detector. The *L*∗ value indicates lightness varying from 0 to 100 (black and white, respectively). The *a*∗ value represents the range from green (−60) to red (+60), while *b*∗ denotes the range from blue (−60) to yellow (+60). The values of *C*∗⁣_ab_ and *h*_ab_ were calculated from *a*∗ and *b*∗ using Equations (5) and (6) [24]:

(5) $$\begin{array}{c} C{*}_{ab}=\sqrt{a^{*2}+b^{*2}}, \end{array}$$

(6) $$\begin{array}{c} h_{ab}=\left(\tan^{-1}\left(\frac{b*}{a*}\right)\times 57.29\right)+180. \end{array}$$

#### 2.4.6. PV and Ultraviolet Absorbance Determinations

The PV (meq O_2_/kg) and conjugated dienes (K_232_) and trienes (K_270_) of the oil samples were determined according to the established European methods described in directive CEE/2568/91 for olive oil [25].

#### 2.4.7. Fatty Acid Analysis

The fatty acid composition of the oil samples was analyzed by gas chromatography–mass spectrometry (GC-MS). Prior to injection, extracted triglycerides were derivatized to fatty acid methyl esters (FAMEs) using the method described by Allay et al. [26]. Analysis was performed on a Thermo GC-MS 1300/TS Q 8000 Evo equipped with aTR-5 capillary column. Injection was performed at 250°C in fractionated mode with helium as the carrier gas (1 mL/min). The oven temperature was programmed in three stages: 120°C–180°C (3°C/min), held for 15 min, then increased to 240°C (5°C/min) and held for 25 min. The FAMEs were then fed into the mass spectrometer, and the peaks were identified using spectra from the NIST database, literature data, and a standard FAME mixture (Sigma-Aldrich). Each sample was analyzed in triplicate, and results were expressed as a percentage of total fatty acids.

#### 2.4.8. Evaluation of OSI

The evaluation of the OSI was conducted using the Rancimat method, measured by the Metrohm Rancimat 743 apparatus (Metrohm Co., Basel, Switzerland) and expressed in induction time (hours). For this analysis, 3 g of oil sample underwent oxidation under conditions involving an airflow of 20 L/h at a temperature of 100°C.

### 2.5. Statistical Analysis

The RSM was conducted using JMP Pro 15 software (SAS Institute Inc., United States). The fitted polynomial equation was represented through 3D surface plots to visually depict the relationship between the response and the experimental levels of each factor. The data were subjected to analysis of variance (ANOVA) analysis and presented in a standardized Pareto chart with a threshold significance of *α* = 5%. The prediction model's efficacy was assessed by comparing the experimental and predicted outcomes. Optimum conditions for extracting maximum yield and high-quality oil were determined and used to further validate the model through triplicate trials.

## 3. Results and Discussion

To assess the effect of three MAE variables (microwave power, ethanol as cosolvent, and extraction time) on hemp seed oil extraction, a BBD was developed with 17 experiments. Fourteen different responses were studied, including oil yield, TPC, tocopherols, OSI, chlorophylls, carotenoids, quality indices (PV and conjugated dienes and trienes), and color parameters (*L*∗, *a*∗, *b*∗, *C*∗⁣_ab_, and *h*_ab_), were detailed in Tables 1, 2, and 3. The linear and quadratic effects of these three variables, as well as their interactions, were examined in depth to optimize microwave extraction conditions and obtain maximum oil yield with high quality.  Table 4 shows the second-order polynomial equations describing predictive models of these responses as a function of power (watts), time (minutes), and ethanol percentage (%EtOH relative to hexane). The ANOVA analysis revealed that 13 prediction models were statistically significant (*p* < 0.05), except the conjugated triene (*λ*270) model, which was not significant (*p* = 0.0975). The correlation coefficients were high (ranging from 0.78 to 0.998), reflecting a good fit of the regression equations to the experimental data. In addition, the lack-of-fit was nonsignificant, indicating that there was a high degree of fit between observed and predicted values in all responses, except the TPC model, which showed a significant (*p* = 0.002) lack of fit but with a very strong *R*^2^ of 0.98.

### 3.1. Extraction Efficiency of Hemp Seed Oil Using MAE

**Figure 1:**
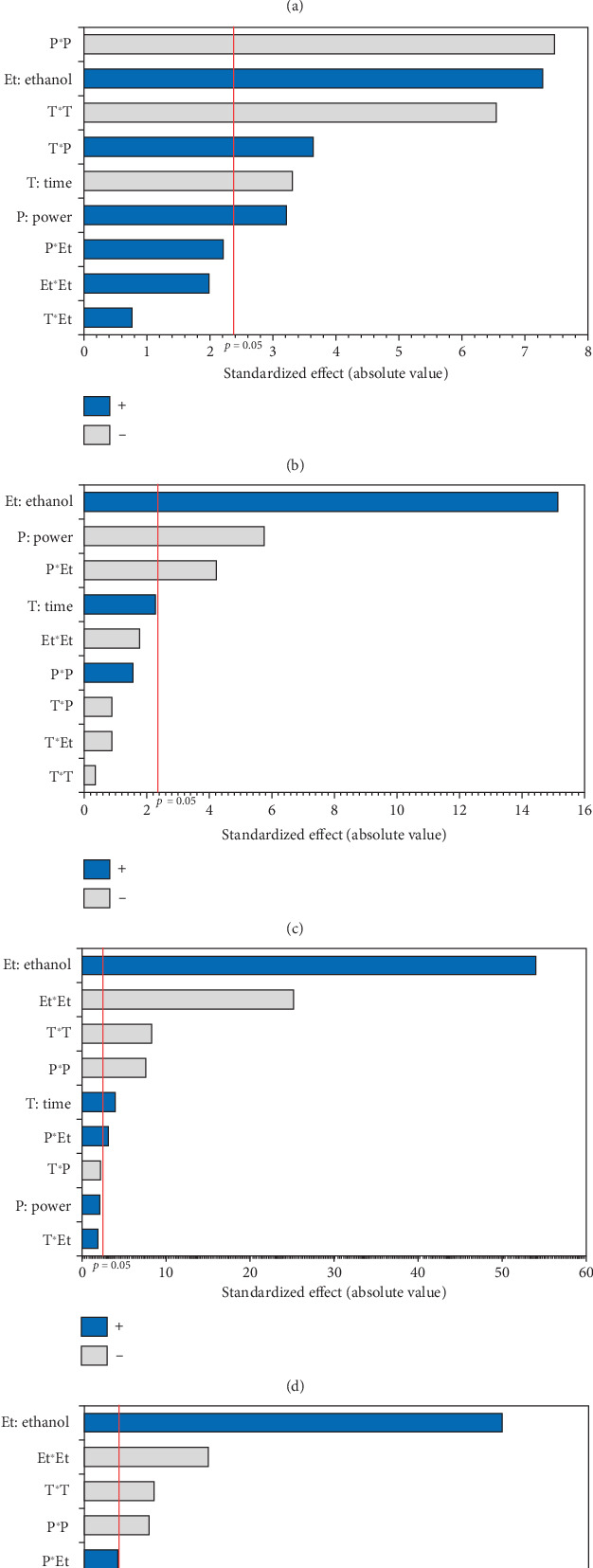
Pareto chart illustrating the effects of variables and their interactions on (a) oil yield, (b) total tocopherols, (c) total phenolic content, (d) total chlorophylls, and (e) total carotenoids.

The experimental results detailed in Table 1 show the variation in oil yield from 26.70% (5 min, 750 W, and 0% EtOH) to 30.38% (10 min, 750 W, and 5% EtOH). Using these experimental results, a second-order polynomial equation was constructed by regression to model the relationship between oil extraction yield and the studied variables (Table 4). An ANOVA was conducted to assess the linear, interaction, and quadratic effects of these three factors on microwave oil extraction efficiency. The results of the ANOVA are summarized in the Pareto chart (Figure 1a). The effects were ranked according to their positive or negative impact on oil yield, with factors having higher coefficients in the equation indicating a greater influence.

According to the Pareto chart, the linear and quadratic effects of the %EtOH, irradiation time, and the quadratic effect of microwave power on oil extraction efficiency were significant (*p* < 0.05). In addition, the linear and quadratic effects of the %EtOH factor had the highest coefficients (1.256 and −1.315, respectively), and therefore, %EtOH was the most important factor in oil extraction efficiency compared to the other factors. The significant interaction effect was observed only for the time and microwave power factors.

**Figure 2:**
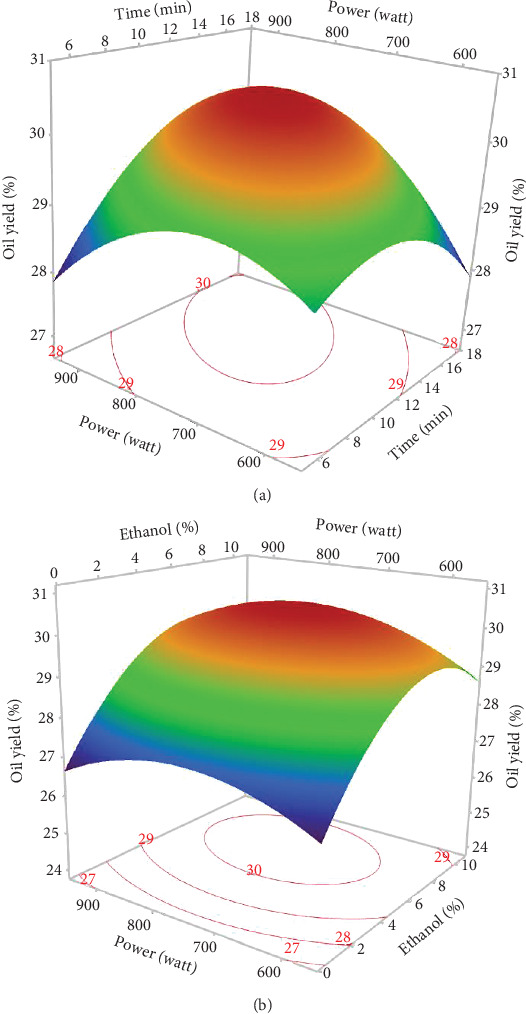
Three-dimensional (3D) response surface and contour plots of different combined effects on the oil yield of hemp seed oil extracted by MAE. Combined effects of (a) microwave power and extraction time and (b) microwave power and ethanol proportion.

The response surface shows a significant increase in oil yield with increasing irradiation time and microwave power (Figure 2). However, beyond 13 min and 750 W, the oil extraction efficiency decreased. Similar trends were observed in the study of Soroush et al. [17] for microwave oil extraction from hemp seeds. This phenomenon can be attributed to the higher temperature with increasing power and time, increasing the permeability of the solvent in the cells and facilitating the diffusion of oil into the solvent [27, 28]. In addition, the application of microwaves can disrupt the cell walls and lipoprotein membranes surrounding fat bodies, promoting oil [29]. On the contrary, rapid temperature changes resulting from excessive microwave power and prolonged extraction times could lead to partial oil degradation, thus reducing the efficiency of microwave extraction [30, 31]. Figure 2 illustrates the general trend of ethanol percentage on oil yield. The yield increased until the ethanol level reached 8%, with a slight reduction at 10%. Therefore, a mixture of hexane and ethanol was more effective for hemp seed oil extraction than pure hexane. As ethanol is a polar solvent with a high dielectric constant, it facilitates the increase in temperature, which increases the oil yield. In addition, using ethanol in MAE solvent mixtures can modify the dielectric constant to reduce energy consumption and extraction time. Studies on microwave extraction of hemp and peony seed oils have also indicated that the use of ethanol, isopropanol, and acetone with hexane improved oil extraction efficiency [17, 32]. However, the limited solubility of the oil in ethanol resulted in a reduction in oil yield at percentages above 8.5%. As observed in the results of Sun et al. [33], MAE of peony seeds using pure ethanol gave the lowest yield compared to hexane and other apolar solvents.

### 3.2. Extraction Efficiency of Tocopherols in Microwave-Extracted Hemp Seed Oil

The experimental results presented in Table 1 show that the tocopherol content varies considerably from 377.10 mg/kg for Run 14 (oil extracted under 600 W for 15 min using 5% EtOH) to 528.27 mg/kg for Run 13 (oil obtained under 900 W for 10 min using 10% EtOH). The ANOVA results, summarized in the Pareto diagram (Figure 1b), indicate that the linear and quadratic effects, as well as the interaction effect of microwave power and irradiation time on tocopherol extraction, were significant. In addition, the power factor had the most significant effect on the extraction of tocopherol from the oil, with the highest quadratic coefficient (−40.791) (Table 4). On the other hand, only the linear effect of the EtOH percentage was significant.

**Figure 3:**
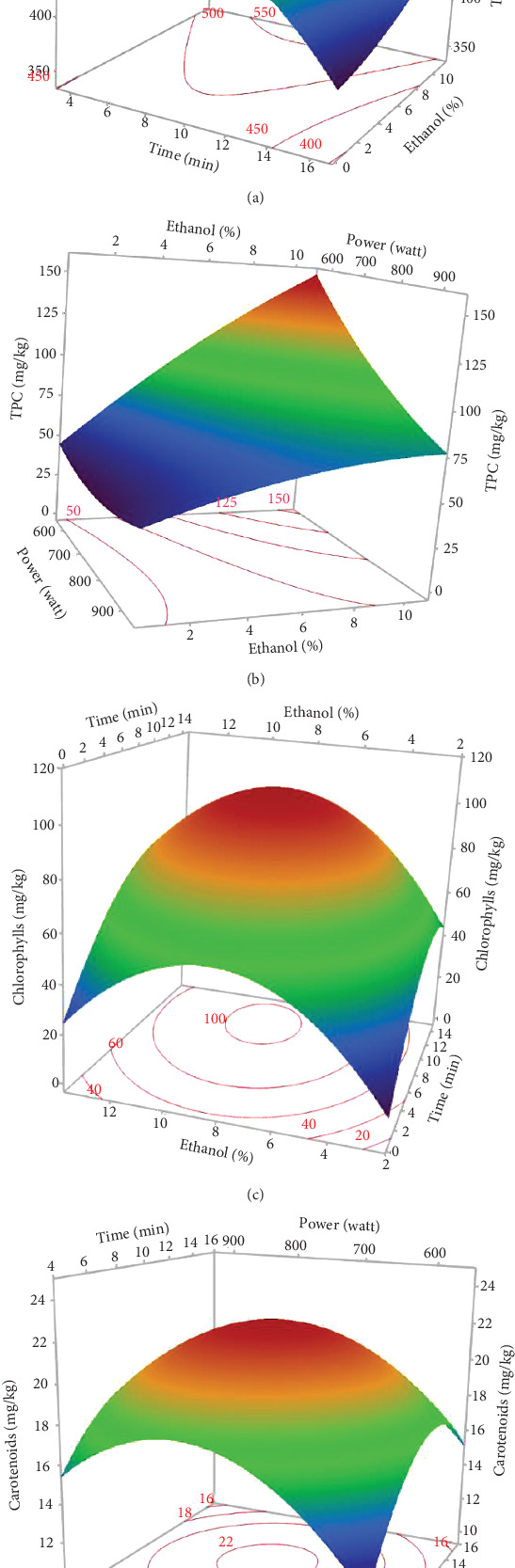
The 3D-surface plots and contour plots of parameters affecting (a) total tocopherols, (b) total phenolic content (TPC), (c) chlorophylls, and (d) carotenoids of MAE hemp seed oil.

The type of solvent is essential for the extraction of bioactive molecules, including tocopherols [34]. As shown in Figure 3a, at fixed microwave powers and extraction times (750 W and 10 min), the oil extracted with pure hexane had the lowest tocopherol content, while the combination with ethanol (up to 10%) produced an oil rich in tocopherols. This may be attributed to the synergistic effect of mixing hexane and ethanol. Plant matrices encompass various classes of bioactive substances, including tocopherols. Their extraction efficiency obviously depends on their individual polarities, which control the degree of their dissolution in a specific solvent [34]. In addition, polar solvents display a notable capacity to absorb microwave energy due to their high dielectric constants, facilitating heat distribution in the sample matrix, thus improving tocopherol extraction efficiency. Similarly, other studies have found that tocopherol levels in oils extracted with polar solvents exceeded those of nonpolar solvents [35].

Increasing microwave power and extraction time led to an increase in tocopherols in hemp seed oil. Tocopherol isomers, in particular, *δ*- and *γ*-tocopherols, absorb microwave energy intensely due to their high dipole moment, leading to an increase in temperature, which facilitates their rapid diffusion in oil [36]. On the other hand, microwaves disrupt the plant cell membranes and contribute to breaking the bond between tocopherols and phospholipids or proteins, allowing them to dissolve in the extraction solvent in a very short time [37]. However, prolonged exposure times (15 min) and high power (900 W) reduced the oil's total tocopherol content. Sundar et al. [9] reported that the lowest levels of *α*-, *δ*-, and *γ*-tocopherols were observed in hemp seed oil exposed to 900 W power for 10 min. This reduction could be attributed to heat-induced oxidation reactions and destruction of heat-sensitive tocopherols [38]. Furthermore, Figure 3a shows that the rate of tocopherol dissolution increases with extraction time due to the extended contact and interaction time between the solvent and the seed powder. However, prolonged exposure time gave unfavorable results, leading to the development of oxidation.

### 3.3. Extraction Efficiency of Phenolic Compounds in the Microwave-Extracted Hemp Seed Oil

#### 3.3.1. TPC

As shown in Table 1, the TPC in the 17 experiments ranged from 30.00 to 143.89 mg GAE/kg oil, respectively, for Run 2 (750 W, 0% EtOH, and 5 min) and Run 6 (600 W, 10% EtOH, and 10 min). As shown in the Pareto diagram (Figure 1c), the positive linear effect of ethanol percentage had the most significant influence on TPC of the extracted oils, which is consistent with the regression coefficient of 34.672, the highest in the second-order equation (Table 4). In addition, the negative linear effect of microwave power significantly affected TPC. No significant quadratic effect was observed (*p* > 0.05), and only the interaction effect between %EtOH and microwave power was significant (*p* < 0.05), displaying a negative effect.

The surface plot presented in Figure 3b displays that the TPC values of microwave-extracted oils increase with increasing proportions of ethanol. Analogous results were found by Soroush et al. [17], indicating that increased proportions of isopropanol (polar solvent) compared to hexane increased phenolic concentrations in microwave-extracted hemp seed oil. The literature suggests that solvent polarity plays a pivotal role in extracting phenolic compounds, given their essentially polar nature [17, 39]. Highly apolar solvents are unsuitable for extracting high levels of phenolic compounds. Thus, the use of a slightly polar mixture of ethanol and hexane creates optimal conditions for their extraction [40]. In addition, polar solvents facilitate heat distribution in the sample matrix thanks to their great capacity to absorb microwave energy (high dielectric constant), thus improving extraction efficiency [17, 40].

The negative linear effect of microwave power is evident in Figure 3b, meaning that an increase in microwave power led to a decrease in TPC. In fact, the highest TPC (143.89 mg GAE/kg) was obtained using 600 W. Our result is consistent with the study of Soroush et al. [17], suggesting that around 540 W is required for maximum phenol extraction in hemp seed oil. Several studies have also concluded that excessive microwave power leads to a reduction in TPC due to the degradation of thermosensitive compounds [41]. In addition, the interaction between %EtOH and microwave power indicated a decrease in TPC. This decrease could be attributed to the degradation of phenolic compounds due to excessive temperature accentuated by ethanol, as it absorbs microwaves effectively (high dielectric constant).

#### 3.3.2. HPLC-DAD/ESI-MS^2^ Analysis of Phenolic Compounds

HPLC-DAD/MS^2^ analysis of the oil samples extracted by MAE under different experimental design conditions revealed the presence of 26 phenolic compounds, including four phenolic acids (two hydroxybenzoic and two hydroxycinnamic acids), five hydroxycinnamic acid amides (HCAAs), and 17 lignanamides (Tables S1 and S2), identified by comparison of the mass of the precursor ions (MS), their fragments (MS^2^) in positive and negative, and their UV-visible spectra with the existing literature [4–6, 22]. In the literature, a few studies have identified and quantified phenolic compounds in hemp seed oil. These studies have shown the presence of other classes of phenolic compounds, such as quercetin, epicatechin, catechin, kaempferol, vitexin, naringenin, and apigenin [7–9].

The quantification results are presented in Table 5, showing that the factors studied, in particular the proportion of %EtOH, play an important role in the absence or presence of phenolic compounds and their content in the oil. The oil sample with the highest phenolic content was extracted at 600 W for 10 min with 10% EtOH, reaching a value of 44.07 mg/kg oil. Under these extraction conditions, the main phenolic compounds detected were benzoic acid (7.33 mg/kg), followed by *p*-hydroxybenzoic acid (3.21 mg/kg), and sinapic acid (2.31 mg/kg), all grouped in the phenolic acid class, which accounted for 30.63% of the detected phenolic compounds. Our results were similar to those observed in the literature, showing that benzoic, *p*-hydroxybenzoic, sinapic, vanillic, ferulic, and *p*-coumaric acids were the main phenolic acids identified in hemp seed oil [42, 43]. In addition, the results highlight the abundance of phenylpropanoids (almost 60% of total phenolic compounds), a phenolic subgroup encompassing HCAAs and lignanamides. The most representative HCAAs were *N*-*trans*-caffeoyltyramine (3.73 mg/kg), followed by *N*-feruloyltyramine (2.88 mg CTE/kg), and *N*-*trans*-coumaroyltyramine (1.51 mg CTE/kg). As for lignanamides, cannabisines A, B, O, and Q were the most representative, with contents of 1.75, 1.75, 1.6, and 1.56 mg CTE/kg, respectively. These observations are in line with numerous earlier studies on hemp seeds that have reported the presence of phenylpropanoids [4, 5, 22]. In addition, phenolic compounds such as *p*-coumaric acid, tri-*p*-coumaroylspermidine, cannabisines (E, M, and Q), 3,3⁣′-demethyl-heliotropamide, and isocannabisin N, as well as the C isomer of cannabisin, the two isomers of cannabisin B, had low levels or were not even detected in most of the extracted oils.

In general, under the same conditions of power and extraction time, the effect of ethanol percentage on phenolic compound extraction in hemp seed oil is very clear. This can be justified by the different polarities of each phenolic compound. As there is a change in polarity in the oil extraction solvent, each compound will be extracted to a greater or lesser extent. Furthermore, the highest levels of phenolic compounds were observed in oils extracted with 10% EtOH, except for isocannabisin N. As mentioned in the TPC assay, increasing the ethanol proportion in the extraction solvent allows for better extraction and solubilization of phenolic compounds in the extracted oil. Regarding the effect of power on phenolic compound extraction, we observed that using high power (up to 900 W) led to a degradation of most phenolic compounds, except for isocannabisin N, sinapic acid, and cannabisin O, which had notable concentrations when applying 900 W. The stability of these compounds at high powers may be due to their resistance to the increase in high temperatures generated by microwaves.

The TPC of the MAE-oils under different conditions, as determined by HPLC-DAD, were relatively low, ranging from 7.51 mg/kg oil for Run 2 (750 W, 0% EtOH and 5 min) to 44.07 mg/kg oil for Run 6 (600 W, 10% EtOH and 10 min), compared to those obtained by the colorimetric method for the same extraction conditions (30–143.89 mg GAE/kg). The likely explanation for the difference between the two methods is the Folin–Ciocalteu method's lack of specificity in measuring phenolic compounds, as it can also react positively with various nonphenolic substances [44]. Consequently, the colorimetric method might result in an overestimation of the TPC.

### 3.4. Extraction Efficiency of Pigments in Microwave-Extracted Hemp Seed Oil

Pigments, including carotenoids and chlorophylls, in hemp seed oils were assessed using MAE, with variations in three factors (power, time, and %EtOH). As indicated in Table 1, the lowest contents of carotenoids (2.59 mg/kg) and chlorophylls (6.72 mg/kg) were observed in the oil extracted under a power of 750 W and 0% EtOH for 5 min (Run 2). In contrast, the highest contents, reaching 99.87 and 28.54 mg/kg (respectively, for chlorophylls and carotenoids), were noted in the oil extracted applying 900 W and 10% EtOH for 10 min (Run 13). The Pareto chart (Figure 1d,e) indicates that the negative quadratic effects of the three factors on the pigment content were significant. In addition, the linear effect of %EtOH and the interaction of %EtOH with microwave power were significant and positive. However, the linear effect of irradiation time was only observed for chlorophylls. The cosolvent percentage was the variable with the greatest effect on pigments, with a regression coefficient of 42.335 for chlorophylls and 11.649 for carotenoids (Table 4).

According to the response surfaces (Figure 3c,d), chlorophyll and carotenoid contents were increased with increasing ethanol percentage, indicating that the solvent combination provides optimal conditions for extracting pigments. However, this increase was limited to around 9% of EtOH, and a relatively small reduction was observed at 10%. This result is in accordance with other studies, which have shown that the highest pigment yield was obtained by adding a more polar solvent to a less polar solvent [45]. The polarity of carotenoids and chlorophylls varies according to their chemical structures and functional groups. Chlorophylls are regarded as more polar molecules, which explains the increase in their extractions with oil by increasing the proportion of ethanol in the extraction solvent. Concerning carotenoids, hemp seeds contain three main carotenoids: lutein being the most abundant, followed by *β*-carotene, and zeaxanthin [46]. Lutein and zeaxanthin are generally more polar molecules, while *β*-carotene is among the least polar carotenoids [47]. This explains why the addition of ethanol to hexane created a moderately polar mixture that enabled the extraction of both apolar and the most polar carotenoids. Our results are in agreement with other studies reporting the efficacy of moderately polar mixtures such as hexane and acetone or hexane and 2-propanol to extract carotenoids and chlorophylls from plant matrices [48, 49]. The decrease in pigment concentration at high ethanol percentages can be explained by the degradation of these compounds with increasing temperature.

Pigment response surfaces indicated that increasing the time and power of microwave irradiation increased chlorophylls and carotenoids. However, longer exposure times and higher power (900 W for 15 min) decreased their content (Figure 3c,d). The same pigment pattern was found by other researchers [9]. Microwaves can penetrate the cells and facilitate their subsequent decomposition as the pigments are dissolved in the extraction solvent. Furthermore, increasing the extraction time allows the powder to have greater contact with the solvent, resulting in the release of more pigments. However, excessive power with longer extraction times can lead to pigment degradation due to high temperatures [29].

### 3.5. Color Parameters of Microwave-Extracted Hemp Seed Oil

The oil color and appearance are parameters of great commercial importance that should be closely monitored to ensure the quality of the final product. The coloration of oils is primarily attributed to the presence of chlorophyll and carotenoid pigments, responsible for the green and yellow color, respectively.  Table 2 presents the color measurement of oils from the 17 experiments using their CIELAB scalar coordinates (*L*∗, *a*∗, and *b*∗), along with other psychophysical characteristics of color, including chromaticity (*C*∗⁣_ab_) and hue angle (*h*_ab_). Notably, all samples are positioned in the second quadrant with negative values of *a*∗ and positive values of *b*∗. Furthermore, there is considerable variation in both *C*∗⁣_ab_ (ranging from 3.76 to 15.7) and *b*∗ values (ranging from 2.58 to 14.57) among the extracted oils. It is noteworthy that the *C*∗⁣_ab_ values align with those corresponding to the *b*∗ values, resulting from the low *a*∗ values (ranging between −2.74 and −6.17). Similar observations have been reported in virgin olive oils [50].

**Figure 4:**
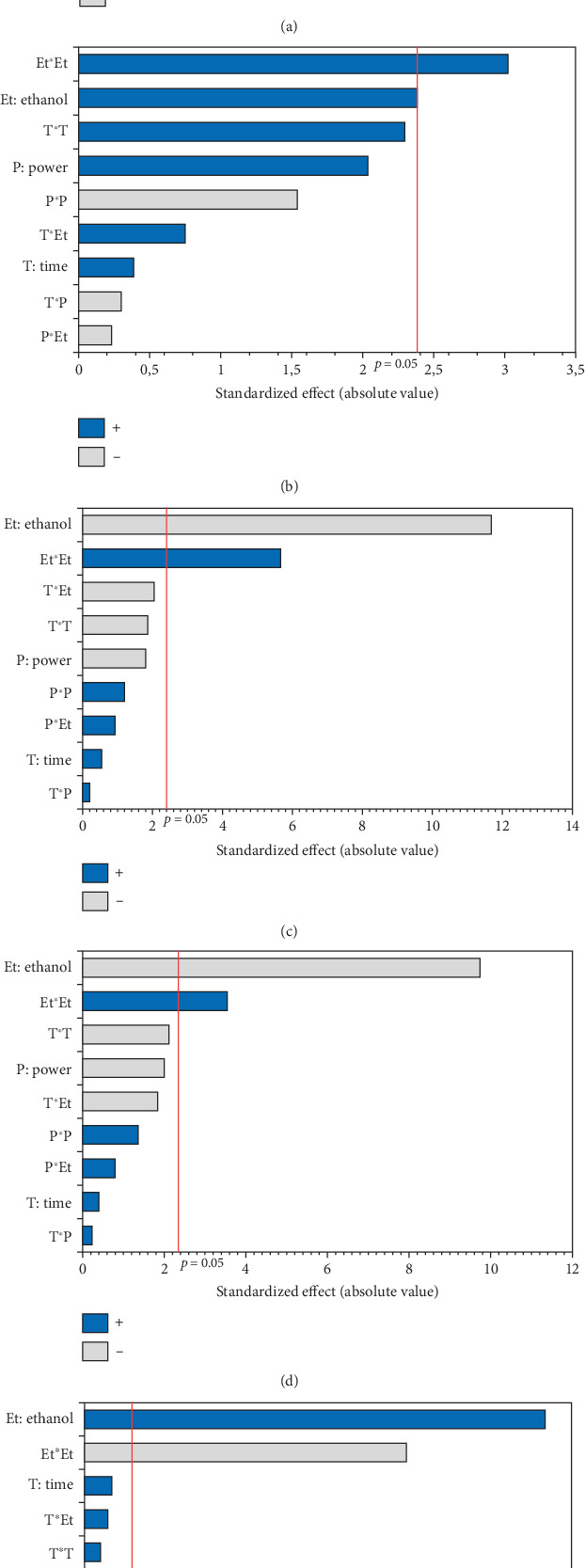
Pareto chart illustrating the effects of variables and their interactions on color parameters of MAE hemp seed oil: (a) *L*∗, (b) *a*∗, (c) *b*∗, (d) *C*∗⁣_ab_, and (e) *h*_ab_.

The *L*∗ values allowed differentiating oils referring to the lightness. Among the results of the 17 experiments, the oil obtained with 5% EtOH, 750 W, and 15 min exhibited the highest *L*∗ value (47.24), indicating a very light color. Conversely, the darkest colored oil (L∗ = 33.74) was obtained under the conditions of 750 W, 10% EtOH, and 5 min. Additionally, the highest *h*_ab_ value (139.38) corresponds to the oil extracted in Run 4 (900 W, 5% EtOH, and 5 min), while the lowest (109.26) was observed in Run 8 (750 W, 5% EtOH, and 10 min). The ANOVA results presented in the Pareto chart (Figures 4a, 4b, 4c, 4d, and 4e) revealed that only the linear and quadratic effects of %EtOH on the color parameters were significant. In addition, the linear effect of extraction time and its interaction with %EtOH were significant for the *L*∗ parameter only.

**Figure 5:**
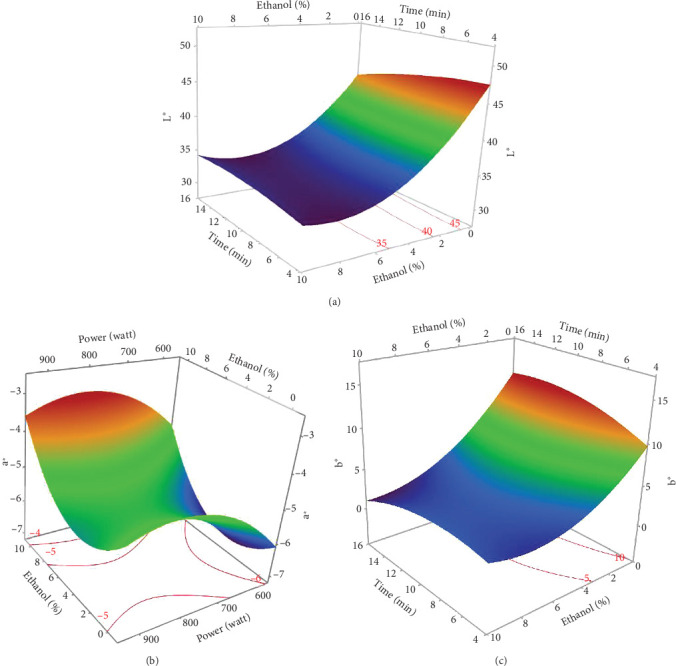
The 3D-surface plots and contour plots of microwave-assisted extraction parameters affecting (a) lightness *L*∗, (b) redness *a*∗, and (c) yellowness *b*∗ of hemp seed oil.

The response surfaces (Figures 5a, 5b, and 5c) of the color parameters show a significant increase for *a*∗ and *h*_ab_ with the increase in the percentage of ethanol. In contrast, a significant decrease was observed for *L*∗, *b*∗, and *C*∗⁣_ab_ with the increase in %EtOH. However, a decrease was observed up to 8% EtOH. According to these response surfaces, the *a*∗ parameter followed a similar trend to the pigment content (chlorophylls and carotenoids), whereas an almost inverse trend was observed for *b*∗ and *L*∗, meaning that the increase in the *a*∗ value could be mainly linked to the presence of chlorophylls in the oil samples, which are responsible for the green color. However, the decrease in the value of *b*∗ despite the increase in the content of carotenoids (responsible for the yellow color) may be due to the high concentration of chlorophylls, hiding the carotenoids' yellow color. As reported for sesame seed [51] and black cumin seed oils [52], the efficient release of chlorophylls and carotenoids from the seeds could be responsible for the changes in oil color parameters (*a*∗, *b*∗, and *L*∗). In addition, other researchers found a very strong positive correlation of pigments with the *a*∗ value and a negative correlation with the *L*∗ value [50], highlighting that pigments are responsible of the intensification of oil color. On the other hand, in a comparative study of oil extracted from roasted and unroasted hemp seeds, the authors showed that the increase in the *b*∗ value in oil extracted from roasted seeds may be due to the decrease in chlorophyll content compared with carotenoids [53].

### 3.6. OSI of Microwave-Extracted Hemp Seed Oil

**Figure 6:**
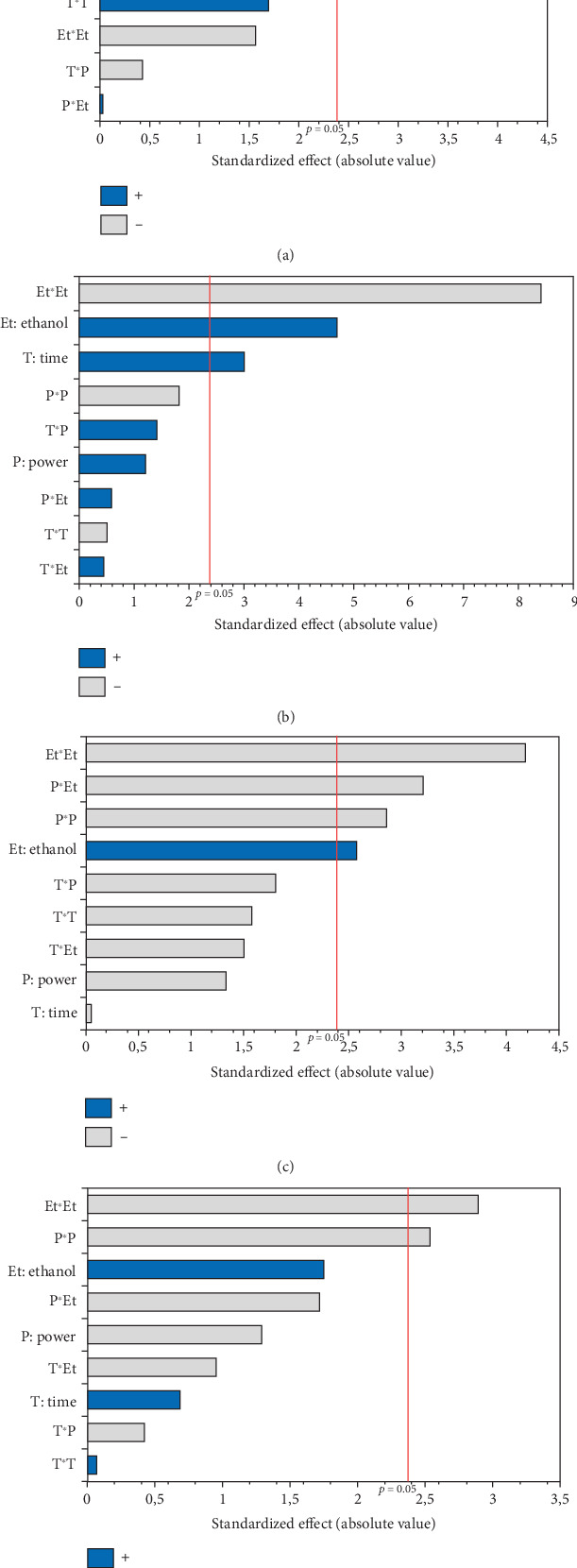
Pareto chart illustrating the effects of variables and their interactions on (a) oxidative stability index, (b) peroxide value, (c) conjugated diene, and (d) conjugated triene of MAE hemp seed oil.

The OSI of microwave-extracted oils was assessed based on induction time using the Rancimat method. As illustrated in Table 3, the induction time ranged from 16.74 (oil extracted at 750 W, 0% EtOH for 15 min) to 34.32 h (oil extracted at 600 W, 5% EtOH for 15 min). The ANOVA results, summarized in the Pareto diagram (Figure 6a), indicate that the linear effect of the three variables on oxidation stability is significant. The effects of irradiation time and %EtOH were positive, while microwave power had a negative impact. In addition, a significant negative interaction was observed between time and %EtOH. According to the second-order equation (Table 4), the percentage of cosolvent had the greatest effect on the OSI of the oil, with a coefficient of 2.75.

**Figure 7:**
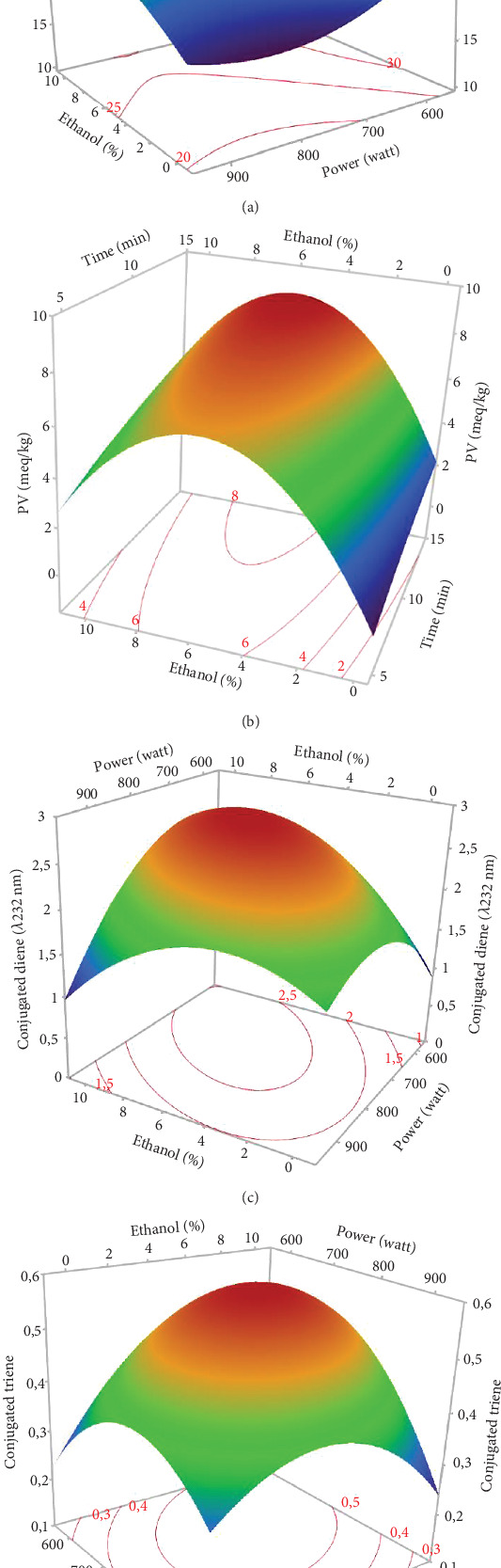
The 3D-surface plots and contour plots of microwave-assisted extraction parameters affecting (a) oxidative stability index (OSI), (b) peroxide value (PV), (c) conjugated diene, and (d) conjugated triene of hemp seed oil.

According to the response surface (Figure 7a), oxidative stability showed a linear improvement with an increase in the percentage of ethanol. This could be mainly associated with the high concentration of bioactive compounds (phenols, tocopherols, and carotenoids) that protect the oil against oxidation. Since antioxidants are moderately polar compounds, the ethanol–hexane combination creates a relatively polar solvent system, offering optimal conditions for their extraction [17, 40]. The positive linear effect of irradiation time suggests that oil stability increases with longer extraction times, which could be related to the increased dissolution of bioactive compounds in the solvent with time [17]. Despite the positive effect of the two independent variables (%EtOH and irradiation time), their interaction had a negative influence on the OSI. Figure 7a shows that a longer exposure time with higher percentages of ethanol gave unfavorable results. This stability decrease might be attributed to the increased temperature during extraction, which leads to the destruction of certain thermolabile antioxidant compounds and the generation of oxidation products that accelerate the oxidation of the oil. The application of high microwave power also contributed to a reduction in the OSI of the extracted oils, as it led to the degradation of antioxidants at high temperatures.

### 3.7. Quality Indices of Microwave-Extracted Hemp Seed Oil

Peroxides are the first oil auto-oxidation products. They are unstable and decompose at high temperatures into aldehydes, ketones, and other stable molecules. In addition, the conjugated dienes (232 nm) and trienes (270 nm) of an oil can reflect its oxidation state. The higher the values at 232 and 270 nm, the more peroxidized the oil [54].

The conjugated dienes and trienes varied among the 17 experiments (1.45–2.82 and 0.3–0.63, respectively). The PV varied considerably from 0.99 to 8.47 meq O_2_/kg oil, respectively, for the oils extracted in Run 12 (900 W, 0% EtOH for 10 min) and Run 10 (750 W, 5% EtOH, and 10 min) (Table 3). The linear and quadratic effects of the %EtOH factor were the most significant on quality indices (PV and conjugated dienes and trienes). The significant quadratic effect of power was observed only for conjugated dienes and trienes, while the linear effect of extraction time was significant only for PV. In addition, the interaction effect between %EtOH and power was significant but only for conjugated dienes (Figures 6b, 6c, and 6d).

The response surfaces of the quality indices show that the PV and conjugated dienes and trienes were relatively increased with increasing %EtOH until 4%, after which a very sharp decrease was observed (Figures 7b, 7c, and 7d). Ethanol absorbs microwave energy effectively due to its high dielectric constant. Consequently, the temperature of the solvent–seed mixture rises and leads to degrading and peroxidizing the extracted oils. In addition, ethanol increases the concentration of chlorophylls, which act as pro-oxidants and accelerate oil degradation. Beyond 4% EtOH, the PV and conjugated dienes and trienes in the oil were greatly reduced. This may be attributed to the high content of bioactive compounds in the extracted oil. Indeed, using a high percentage of ethanol to extract oil results in extracting large quantities of phenolic compounds, tocopherols, and carotenoids. In agreement with our results, the PV of the oil extracted from hemp seeds decreases as the proportion of polar solvent (isopropanol) in the extraction solvent mixture (hexane–isopropanol) increases [17].

According to Figure 7b, extending the extraction duration from 5 to 15 min increased the oil's PV. This rise in PV can be attributed to prolonged oil exposure to high temperatures during the extraction process, leading to increased oxidation and active radical generation [16]. Similar findings were reported by Soroush et al. [17], who observed an increase in PV with extended extraction time and higher microwave power. Regarding the notable impact of power, it was specifically observed for conjugated dienes and trienes. The response surface plots indicated that an initial increase in microwave power from 600 to 700 W led to a rise in conjugated dienes and trienes due to the temperature elevation induced by microwaves (Figure 7c,d). This could be ascribed to higher microwave power that increased solvent temperature, promoting the breakdown of initial oxidation products (hydroperoxides) into secondary products like malondialdehydes. Additionally, the high microwave power causes phenolic compound degradation [16]. Other studies have similarly concluded that excessive microwave power causes the decomposition of primary products into secondary ones, resulting in an increase in the secondary oxidation index [17]. Furthermore, the significant negative interaction term between power and %EtOH indicates that, aside from the temperature increase induced by microwave power, ethanol absorbs microwave energy due to its high dielectric constants. This excessive temperature rise may contribute to the conversion of primary oxidative compounds (peroxides) into secondary oxidative molecules (ketones and aldehydes), potentially explaining the decrease in conjugated dienes in hemp seed oil.

### 3.8. Optimizing MAE of Hemp Seed Oil

To optimize the extraction parameters, RSM was employed. The optimization process was aimed at maximizing oil yield while also considering the content of bioactive compounds. The optimal extraction conditions were selected not only for achieving the highest oil yield but also because they corresponded to near-maximum levels of key bioactive compounds, particularly total tocopherols and carotenoids, while ensuring satisfactory phenolic content and good oxidative stability of the oil.

Based on this approach, the optimal extraction conditions for hemp seed oil using MAE were established at 13.60 min extraction time, 800 W power, and 7.5% ethanol concentration. Under these extraction conditions, oil yield, TPC, tocopherols, OSI, total chlorophylls and carotenoids, quality index (PV and conjugates [diene and triene]) *L*∗, *a*∗, *b*∗, *C*∗⁣_ab_ and *h*_ab_ were 30.67%, 86.11 mg GAE/kg oil, 506.67 mg/kg, 26.67 h, 98.18 mg/kg 26.17 mg/kg, 8.55 mg O_2_/kg, 2.37, 0.54 and 33.30, −4.15, 2.74, 4.71, and 140.55, respectively. The accuracy of the predictive model was confirmed through experimental validation at the optimal point. As shown in Table 6, the corresponding experimental values for oil yield, TPC, total tocopherols, OSI, total chlorophylls and carotenoids, PV, conjugates (diene and triene) *L*∗, *a*∗, and *b*∗ were 88.55 mg GAE/kg, 510.64 mg/kg, 28.60 h, 99.68 and 27.31 mg/kg, 7.68 mg O_2_/kg, 2.24 and 0.51, 33.54, −4.01, 3.17, 5.11, and 141.72, respectively. Therefore, the RSM-based MAE optimization was satisfactory, and the predictive models were successfully validated.

Two studies applying a design of experiment and RSM approach to hemp seed oil extraction have been identified in the literature. Soroush et al. [17] studied the effect of microwave power, irradiation time, and solvent ratio on the yield and quality of the extracted oil. They determined that the optimum conditions were a power of 450 W and a hexane:isopropanol ratio of 3/2 (*v*/*v*) for 1 min, giving a maximum yield of 25.76%. This yield is lower than that obtained in our study, where optimization achieved a higher yield. On the other hand, Rezvankhah et al. [16], using the MAE with a power of 450 W for 7.19 min, obtained an oil yield of 33.91%, higher than our result. These differences can be attributed to several factors, including the variety of hemp seeds used, the specific extraction conditions, the type of solvent employed, and the pedoclimatic conditions, which significantly influence the quality and quantity of oil extracted. Taaifi et al. [3] have highlighted the impact of varietal variations and climatic conditions on the oil yield of different hemp seed varieties.

In our study, optimal conditions enabled us to extract an oil rich in bioactive molecules, with a total TPC of 88.55 mg GAE/kg and a tocopherol concentration of 510.64 mg/kg. These antioxidants play a crucial role in preventing lipid oxidation, giving the oil notable oxidative stability (28.6 h) and satisfactory quality, characterized by a PV of 7.68 meq O_2_/kg oil and conjugated diene and triene values of 0.51 and 2.239, respectively. Indeed, tocopherols, as powerful antioxidants, are essential for preserving oil quality and preventing rapid lipid degradation, which partly explains the good oxidative stability of the oil obtained in our study.

However, in comparison with our results, Soroush et al. [17] reported much higher TPC values, reaching 3910 mg GAE/kg, while Rezvankhah et al. [16] observed higher tocopherol concentrations, reaching 929.67 mg/kg under their respective optimal conditions. These discrepancies may be explained by varietal differences, specific pedoclimatic conditions, and particular extraction parameters, as suggested by Irakli et al. [46] and Taaifi et al. [3]. Furthermore, the PV obtained in these studies was lower than ours: Soroush et al. [17] reported a maximum value of 3.87 meq O_2_/kg for extraction at 450 W for 1 min with a hexane:isopropanol mixture, while Rezvankhah et al. [16] observed a value of 2.50 meq O_2_/kg under 450 W for 7.19 min. This difference could be attributed to the more intense extraction conditions in our study (800 W for 13.60 min), with higher power and prolonged extraction time favoring lipid oxidation and degradation. In addition, the high TPC and tocopherol content reported in these two studies could also explain their lower PV, as these compounds are known to play an essential role in protecting against lipid oxidation. The tocopherol family is the main source of antioxidants in hemp seed oil. Among these tocopherols, gamma-tocopherols play a particularly crucial role in the oil's stability, thanks to their unique antioxidant properties. This isomer is essential for protecting the oil against oxidation and preventing the formation of hydroperoxides, unstable compounds that can impair oil quality and shorten its shelf life [2].

As far as pigments are concerned, the carotenoid and chlorophyll contents of the oil extracted under our optimum conditions were 27.31 and 99.68 mg/kg, respectively, close to the maximum values observed in the experiment (28.80 and 99.87 mg/kg, respectively, in Run 13). These concentrations strongly influence the coloration of the oil obtained. Indeed, the oil extracted under our optimal conditions showed low colorimetric values for *L*∗ (33.54), *b*∗ (3.17), and *C*∗⁣_ab_ (5.11), while values for *a*∗ and *h*_ab_ were high (−4.01 and 141.72, respectively). As previously mentioned, an increase in *a*∗ and a decrease in *b*∗ and *L*∗ are mainly associated with a high concentration of chlorophylls, which impart a dominant green hue to the oil, masking the yellow coloration of the carotenoids. These observations are in line with those of Rabadán et al. [55], who also highlighted a correlation between color parameters and the presence of chlorophyll and carotenoid pigments in different vegetable oils.

### 3.9. Fatty Acid Profile of Hemp Oil Extracted by MAE Under Optimal Conditions

The fatty acid profile of hemp seed oil extracted by MAE under optimal conditions was analyzed using GC-MS (Table 6). The oil exhibited a high proportion of polyunsaturated fatty acids (65.30%), followed by monounsaturated fatty acids (18.41%) and saturated fatty acids (15.10%). Linoleic acid constituted the majority at 50.23%, followed by oleic acid (18.23%), *α*-linolenic acid (14.09%), palmitic acid (10.30%), and stearic acid (3.09%). Minor fatty acids included *γ*-linolenic acid (0.98%), arachidic acid (0.62%), behenic acid (0.34%), eicosenoic acid (0.18%), and lignoceric acid (0.13%). The well-balanced omega-6/omega-3 ratio of 3.65 is in line with nutritional recommendations for a healthy diet [56].

This profile is consistent with solvent-extracted hemp oils from the same variety studied [3, 21]. However, Rezvankhah et al. [16], who also studied MAE-extracted hemp oil, reported higher proportions of linoleic acid (55%) and *α*-linolenic acid (18%), with slightly lower levels of oleic acid (15.98%) and *γ*-linolenic acid (0.6%). They noted that this profile remained comparable to Soxhlet-extracted oils. These differences may arise from genetic differences (e.g., cultivar-specific lipid biosynthesis) or climatic factors (e.g., temperature and soil conditions), which are well-documented influencers of fatty acid composition in hemp seeds [3, 46].

## 4. Conclusion

The MAE was employed to extract oil from hemp seeds. The optimal conditions for microwave-assisted oil extraction were determined using RSM. A BBD was implemented to assess the impact of three variables: extraction time, microwave power, and the percentage of ethanol on various parameters such as oil extraction efficiency, TPC, tocopherols, OSI, chlorophylls, carotenoids, quality indices (PV and conjugates [diene and triene]), and color (*L*∗, *a*∗, *b*∗, *C*∗⁣_ab_, and *h*_ab_). The statistical analysis revealed that the linear and quadratic effects of the %EtOH variable exerted the most significant influence on all responses. Additionally, an HPLC-DAD/MS^2^ analysis conducted on the oils extracted by MAE indicated that the content of phenolic compounds in the extracted oils was highly influenced by the ethanol and microwave power. The responses were optimized to maximize yield. The optimal conditions identified were 800 W using 7.5% EtOH for 13.60 min. Under these conditions, a maximum yield of 30.69% was achieved, facilitating the extraction of an oil rich in bioactive compounds (phenols and tocopherols) with acceptable oxidation quality. Although the MAE of hemp seed oil has been successful, the use of hexane as an oil extraction solvent is increasingly diminishing due to its toxicity and environmental impacts. This invites us to look for alternative solvents that are less toxic and environmentally friendly for extracting good quality oil without affecting the yield.

## Figures and Tables

**Table 1:** Experimental results of oil yield, total phenolic content, tocopherols, chlorophylls, and carotenoids of hemp seed oils extracted by microwave-assisted extraction under various experimental conditions.

**Run**	**Independent variables**			**Responses**				
	**Time (min)**	**Power (W)**	**Ethanol (%)**	**Oil yield**	**TPC**	**Total tocopherols**	**Total chlorophylls**	**Total carotenoids**
1	5	600	5	29.07 ± 0.10	77.81 ± 2.54	449.33 ± 3.07	63.28 ± 2.85	17.05 ± 0.94
2	5	750	0	26.70 ± 0.05	30.00 ± 1.91	472.41 ± 3.97	6.72 ± 2.63	2.59 ± 0.09
3	5	750	10	29.39 ± 0.38	98.19 ± 1.70	518.15 ± 56.04	86.68 ± 3.38	25.10 ± 0.01
4	5	900	5	28.01 ± 0.17	66.72 ± 1.70	447.42 ± 13.89	67.65 ± 1.34	17.66 ± 0.61
5	10	600	0	26.99 ± 0.23	40.34 ± 2.97	455.46 ± 62.37	7.92 ± 0.77	4.11 ± 0.3
6	10	600	10	29.13 ± 0.78	143.89 ± 2.76	491.66 ± 25.06	85.98 ± 2.34	24.33 ± 1.06
7	10	750	5	30.03 ± 0.12	76.16 ± 1.48	501.86 ± 10.48	85.68 ± 0.12	22.07 ± 0.66
8	10	750	5	29.87 ± 0.12	73.91 ± 1.70	514.88 ± 21.08	84.66 ± 1.44	23.19 ± 1.56
9	10	750	5	30.18 ± 0.42	73.61 ± 0.85	523.70 ± 24	88.07 ± 1.73	23.01 ± 0.47
10	10	750	5	30.38 ± 0.21	76.91 ± 3.39	497.2 ± 9.78	85.14 ± 1.16	22.77 ± 0.31
11	10	750	5	30.28 ± 0.31	73.76 ± 4.03	508.75 ± 18.08	85.26 ± 5.74	22.15 ± 1.49
12	10	900	0	27.32 ± 0.44	31.80 ± 1.91	442.43 ± 6.77	8.04 ± 0.60	3.21 ± 0.02
13	10	900	10	29.88 ± 0.41	80.35 ± 1.06	528.27 ± 25.57	99.87 ± 2.14	28.80 ± 1.28
14	15	600	5	29.52 ± 0.20	96.39 ± 2.54	377.10 ± 18.14	74.56 ± 2.52	17.84 ± 0.18
15	15	750	0	27.40 ± 0.87	43.64 ± 3.81	442.44 ± 13.92	8.14 ± 0.36	3.67 ± 0.07
16	15	750	10	30.06 ± 0.08	100.73 ± 3.18	505.18 ± 15.64	96.97 ± 0.75	28.54 ± 0.01
17	15	900	5	30.20 ± 0.19	73.61 ± 1.27	457.29 ± 17.29	69.42 ± 5.29	16.35 ± 0.26

*Note: *Mean ± SD of three independent experiments (*n* = 3). Oil yield is expressed in grams per 100 g of seeds (percent). Total phenolic content (TPC) is expressed in milligram gallic acid equivalent per kilogram of oil. Total tocopherols, carotenoids, and chlorophylls are expressed in milligrams per kilogram of oil.

**Table 2:** Experimental results of color parameters (*L*∗, *a*∗, *b*∗, *C*∗⁣_ab_, and *h*_ab_) of microwave-extracted hemp seed oils under various conditions.

**Run**	**Independent variables**			**Responses**				
	**Time (min)**	**Power (W)**	**Ethanol (%)**	**L**∗	**a**∗	**b**∗	**C**∗⁣_**ab**_	**h**_**a** **b**_
1	5	600	5	35.65 ± 0.18	−6.17 ± 0.10	6.21 ± 0.15	8.75 ± 0.18	112.60 ± 2.88
2	5	750	0	47.24 ± 0.62	−3.91 ± 0.01	9.48 ± 1.31	10.26 ± 1.2	135.00 ± 0.00
3	5	750	10	33.74 ± 0.03	−2.74 ± 0.01	2.58 ± 0.02	3.76 ± 0.02	138.33 ± 10
4	5	900	5	34.92 ± 0.07	−4.71 ± 0.21	4.71 ± 0.21	6.66 ± 0.30	139.38 ± 0.41
5	10	600	0	45.12 ± 0.16	−5.38 ± 0.01	14.75 ± 0.07	15.70 ± 0.06	133.97 ± 0.16
6	10	600	10	34.76 ± 0.11	−4.74 ± 0.14	4.48 ± 0.12	6.52 ± 0.19	110.95 ± 0.55
7	10	750	5	36.12 ± 0.07	−5.83 ± 0.03	6.05 ± 0.06	8.40 ± 0.07	110.05 ± 0.14
8	10	750	5	35.18 ± 0.01	−4.91 ± 0.04	4.78 ± 0.01	6.85 ± 0.04	109.26 ± 0.09
9	10	750	5	35.06 ± 0.24	−5.06 ± 0.01	4.83 ± 0.09	6.99 ± 0.07	136.73 ± 0.16
10	10	750	5	34.83 ± 0.23	−4.72 ± 0.01	4.47 ± 0.06	6.50 ± 0.03	136.65 ± 0.09
11	10	750	5	35.35 ± 0.13	−5.66 ± 0.13	5.48 ± 0.13	7.87 ± 0.18	135.80 ± 0.21
12	10	900	0	46.64 ± 0.81	−4.67 ± 0.28	12.18 ± 0.37	13.04 ± 0.44	136.97 ± 0.07
13	10	900	10	34.52 ± 0.12	−4.32 ± 0.09	3.98 ± 0.07	5.87 ± 0.12	136.37 ± 0.47
14	15	600	5	35.08 ± 0.01	−5.10 ± 0.09	4.54 ± 0.08	6.82 ± 0.02	137.32 ± 0.10
15	15	750	0	43.88 ± 0.01	−4.92 ± 0.05	14.07 ± 0.07	14.90 ± 0.08	136.60 ± 0.49
16	15	750	10	33.82 ± 0.19	−2.79 ± 0.01	2.60 ± 0.00	3.81 ± 0.01	134.84 ± 0.23
17	15	900	5	34.59 ± 0.02	−4.02 ± 0.05	3.45 ± 0.09	5.29 ± 0.10	135.96 ± 0.06

*Note:*Mean ± SD of three independent experiments (*n* = 3).

**Table 3:** Experimental results of peroxide value (PV), conjugated (diene and triene), and oxidative stability index (OSI) of hemp seed oils under various conditions for the extraction of hemp seed oil by microwave-assisted extraction.

**Run**	**Independent variables**			**Responses**			
	**Time (min)**	**Power (W)**	**Ethanol (%)**	**Extinction coefficients**		**PV (meq O**_**2**_ **/kg oil)**	**OSI (hours)**
				**Conjugated diene (** ***λ*** ** 232 nm)**	**Conjugated triene (** ***λ*** ** 270 nm)**		
1	5	600	5	1.98 ± 0.01	0.47 ± 0.02	4.98 ± 0.04	25.43 ± 1.55
2	5	750	0	1.64 ± 0.02	0.36 ± 0.04	1.97 ± 0.01	16.74 ± 0.71
3	5	750	10	2.63 ± 0.11	0.62 ± 0.10	4.42 ± 0.62	28.78 ± 0.00
4	5	900	5	2.42 ± 0.09	0.52 ± 0.00	5.91 ± 0.05	23.99 ± 0.69
5	10	600	0	1.77 ± 0.03	0.41 ± 0.01	2.00 ± 0.00	26.20 ± 0.24
6	10	600	10	2.60 ± 0.04	0.54 ± 0.00	4.92 ± 0.04	32.65 ± 1.14
7	10	750	5	2.50 ± 0.04	0.63 ± 0.01	7.46 ± 0.77	24.23 ± 0.78
8	10	750	5	2.80 ± 0.04	0.56 ± 0.05	7.36 ± 0.66	23.00 ± 1.61
9	10	750	5	2.82 ± 0.02	0.57 ± 0.06	7.43 ± 0.76	25.76 ± 1.24
10	10	750	5	2.68 ± 0.02	0.56 ± 0.07	8.47 ± 0.75	24.95 ± 1.38
11	10	750	5	2.74 ± 0.00	0.59 ± 0.02	8.40 ± 0.57	25.88 ± 0.86
12	10	900	0	2.02 ± 0.11	0.39 ± 0.00	0.99 ± 0.01	17.95 ± 0.67
13	10	900	10	1.45 ± 0.03	0.30 ± 0.01	4.91 ± 0.01	24.51 ± 0.67
14	15	600	5	2.45 ± 0.04	0.50 ± 0.02	6.42 ± 0.74	34.32 ± 0.63
15	15	750	0	1.88 ± 0.11	0.48 ± 0.01	2.93 ± 1.36	28.58 ± 0.42
16	15	750	10	2.22 ± 0.08	0.55 ± 0.03	5.99 ± 0.02	25.53 ± 0.49
17	15	900	5	2.09 ± 0.09	0.49 ± 0.01	9.87 ± 0.04	31.08 ± 1.52

*Note:*Mean ± SD of three independent experiments (*n* = 3).

**Table 4:** Analysis of variance (ANOVA) and regression equation coefficients for each response studied of hemp seed oil extraction by microwave-assisted extraction.

**Response (** **Y** **)**	**β**_0_	**β**_1_	**β**_2_	**β**_3_	**β**_12_	**β**_13_	**β**_23_	**β**_11_	**β**_22_	**β**_33_	**p** ** value**		**R** ^2^	**R** ^2^ ** adj**
											**Model**	**Lack of fit**		
Yield	30.148	0.501	0.0875	1.256	0.435	−0.0075	0.105	−0.445	−0.502	−1.315	0.0001	0.0981	0.97	0.94
TPC	74.87	5.206	−13.243	34.672	−2.922	−2.775	−13.75	−1.096	4.858	−5.633	< 0.0001	0.002	0.98	0.95
Tocopherols	509.278	−13.162	12.732	28.815	20.525	4.25	12.41	−35.701	−40.791	10.968	0.0003	0.378	0.97	0.965
OSI	24.764	3.071	−2.633	2.75	−0.45	−3.772	0.028	1.760	2.180	−1.617	0.0082	0.0564	0.90	0.78
Carotenoids	−22.424	0.497	0.337	11.649	−0.523	0.591	1.343	−2.668	−2.531	−4.780	< 0.0001	0.1799	0.993	0.984
Chlorophylls	85.762	3.095	1.655	42.335	−2.378	2.220	3.442	−8.930	−8.103	−27.205	< 0.0001	0.0744	0.998	0.995
Color														
*L*∗	35.308	−0.523	0.008	−5.76	0.06	0.86	−0.44	−0.419	0.171	4.781	< 0.0001	0.224	0.993	0.983
*a*∗	−5.236	0.088	0.458	0.536	−0.095	0.24	−0.073	0.712	−0.475	0.934	0.039	0.1791	0.80	0.532
*b*∗	5.122	0.21	−0.708	−4.605	0.103	−1.143	0.518	−1.03	0.635	3.090	0.0003	0.0599	0.96	0.914
*C*∗⁣_ab_	7.322	0.174	−0.866	−4.242	0.14	−1.148	0.503	−1.271	0.829	2.132	0.0012	0.0927	0.95	0.875
*h*_ab_	−0.772	0.0125	0.005	0.228	0.005	0.015	0.0001	0.011	0.006	−0.219	< 0.0001	0.1090	0.99	0.977
Quality indices														
Conjugated diene (*λ*232)	2.709	−0.004	−0.1038	0.199	−0.198	−0.165	−0.351	−0.169	−0.304	−0.446	0.0131	0.0685	0.89	0.75
Conjugated triene (*λ*270)	0.571	0.015	−0.029	0.039	−0.013	−0.029	−0.052	0.002	−0.075	−0.086	0.0975	0.1132	0.78	0.5
Peroxide value (PV)	21.156	1.651	0.645	2.551	1.093	0.35	0.438	−0.383	−1.3655	−6.313	0.0016	0.0809	0.94	0.864

*Note: Y* = *β*_0_ + *β*_1_*x*_1_ + *β*_2_*x*_2_ + *β*_3_*x*_3_ + *β*_12_*x*_1_*x*_2_ + *β*_13_*x*_1_*x*_3_ + *β*_23_*x*_2_*x*_3_ + *β*_11_*x*_1_^2^ + *β*_22_*x*_2_^2^ + *β*_33_*x*_3_^2^, where *x*_1_ is the time (minutes); *x*_2_ is the power of microwaves (watts); *x*_3_ is the ethanol; *β*_0_ is a constant; *β*_1_, *β*_2_, and *β*_3_ are the linear coefficients; *β*_12_, *β*_13_, and *β*_23_ are the interaction coefficients; and *β*_11_, *β*_22_, and *β*_33_ are the quadratic coefficients.

**Table 5:** Effect of different conditions (power, time, and %EtOH) on the phenolic composition of hemp seed oil extracted by microwave-assisted extraction.

**Phenolic compounds**	**600 W**				**750 W**					**900 W**			
	**5 min**	**10 min**		**15 min**	**5 min**		**10 min**	**15 min**		**5 min**	**10 min**		**15 min**
	**5% EtOH**	**0% EtOH**	**10% EtOH**	**5% EtOH**	**0% EtOH**	**10% EtOH**	**5% EtOH**	**0% EtOH**	**10% EtOH**	**5% EtOH**	**0% EtOH**	**10% EtOH**	**5% EtOH**
Phenolic acids													
*p*-Hydroxybenzoic acid	1.46 ± 0.08	0.51 ± 0.05	3.21 ± 0.11	1.7 ± 0.03	0.52 ± 0.00	1.97 ± 0.01	1.53 ± 0.02	0.56 ± 0	2.23 ± 0.08	1.36 ± 0.06	0.56 ± 0.02	1.61 ± 0.03	1.21 ± 0.05
Benzoic acid	2.83 ± 0.11	0.53 ± 0.01	7.33 ± 0.29	4.16 ± 0.17	0.50 ± 0.02	4.17 ± 0.01	3.27 ± 0.05	0.67 ± 0.02	5.16 ± 0.18	3.43 ± 0.12	0.63 ± 0.01	4.32 ± 0.03	3.37 ± 0.26
*p*-Coumaric acid	0.38 ± 0.01	0.24 ± 0.02	0.63 ± 0.00	0.49 ± 0.01	0.23 ± 0.00	0.46 ± 0.00	0.41 ± 0.00	0.25 ± 0.01	0.52 ± 0.02	0.44 ± 0.01	0.24 ± 0.01	0.51 ± 0.01	0.45 ± 0.02
Sinapic acid	3.59 ± 0.40	2.62 ± 0.42	2.31 ± 0.35	1.67 ± 0.04	2.89 ± 0.25	3.49 ± 0.63	2.31 ± 0.06	3.14 ± 0.18	3.22 ± 0.84	2.09 ± 0.24	1.52 ± 0.10	3.14 ± 0.68	3.25 ± 0.03
Total phenolic acids	8.26	3.9	13.48	8.02	4.14	10.09	7.52	4.62	11.13	7.28	2.95	9.58	8.28

Hydroxycinnamic acid amides (HCAAs)^a^													
*N-trans*-caffeoyltyramine isomer	0.43 ± 0.01	Nd	1.85 ± 0.07	0.98 ± 0.02	Nd	0.96 ± 0.01	0.56 ± 0.00	Nd	1.18 ± 0.02	0.56 ± 0.02	Nd	0.93 ± 0.01	0.52 ± 0.03
*N-trans*-caffeoyltyramine	0.92 ± 0.03	Nd	3.73 ± 0.15	0.76 ± 0.02	Nd	2.26 ± 0.01	0.84 ± 0.03	Nd	2.88 ± 0.09	0.43 ± 0.02	Nd	0.44 ± 0.01	0.47 ± 0.01
*N*-*trans*-coumaroyltyramine	Nd	Nd	1.51 ± 0.07	1.09 ± 0.02	Nd	0.87 ± 0.02	Nd	Nd	1.01 ± 0.05	0.50 ± 0.02	Nd	0.54 ± 0.01	Nd
*N*-feruloyltyramine	1.16 ± 0.07	0.22 ± 0.01	2.88 ± 0.09	1.71 ± 0.06	0.42 ± 0.04	1.86 ± 0.04	1.04 ± 0.02	0.49 ± 0.01	2.27 ± 0.06	0.73 ± 0.06	0.44 ± 0.01	0.83 ± 0.04	0.52 ± 0.01
Tri-*p*-coumaroylspermidine	Tr	Tr	Tr	Tr	Nd	Tr	Tr	Nd	Nd	Nd	Nd	Nd	Nd
Total HCAAs	2.51	0.22	9.97	4.54	0.42	5.95	2.44	0.49	7.34	2.26	0.44	2.74	1.51

Lignanamides^a^													
*N*-caffeoyltyramine dimer	0.43 ± 0.01	Nd	0.62 ± 0.01	0.67 ± 0.02	Nd	0.47 ± 0.01	0.46 ± 0.01	Nd	0.50 ± 0.01	0.4 ± 0.02	Nd	0.50 ± 0.02	0.47 ± 0.06
Cannabisin A	0.47 ± 0.01	Nd	1.75 ± 0.11	0.42 ± 0.01	Nd	1.07 ± 0.07	0.49 ± 0.02	Nd	1.43 ± 0.05	Nd	Nd	0.38 ± 0.01	Nd
Cannabisin B	0.48 ± 0.02	Nd	1.75 ± 0.12	0.40 ± 0.01	Nd	1.12 ± 0.05	0.47 ± 0.02	Nd	1.45 ± 0.04	Nd	Nd	0.37 ± 0.01	Nd
Cannabisin B isomer 1	0.51 ± 0.01	Nd	1.4 ± 0.04	1.35 ± 0.14	Nd	1 ± 0.00	0.47 ± 0.00	Nd	1.23 ± 0.03	Nd	Nd	0.34 ± 0.01	Nd
Cannabisin B isomer 2	Tr	Tr	Tr	Tr	Nd	Tr	Tr	Nd	Nd	Nd	Nd	Nd	Nd
Demethylgrossamide	0.13 ± 0.19	Nd	0.96 ± 0.03	0.55 ± 0.05	Nd	0.62 ± 0.06	0.21 ± 0.29	Nd	0.85 ± 0.02	Nd	Nd	Nd	Nd
Cannabisin C	0.40 ± 0.01	Nd	0.60 ± 0.01	0.42 ± 0.03	Nd	0.49 ± 0.01	0.48 ± 0.01	Nd	0.53 ± 0.03	0.41 ± 0.01	Nd	0.44 ± 0.02	Nd
3.3-Didemethylgrossamide	0.42 ± 0.001	Nd	1 ± 0.08	0.59 ± 0.05	Nd	0.60 ± 0.002	0.41 ± 0.02	Nd	0.76 ± 0.05	Nd	Nd	Nd	Nd
Cannabisin E	0.51 ± 0.02	Nd	1.12 ± 0.06	0.57 ± 0.01	Nd	0.61 ± 0.01	0.58 ± 0.02	sNd	0.77 ± 0.03	0.72 ± 0	Nd	0.85 ± 0.03	0.71 ± 0.05
Cannabisin M	0.18 ± 0.25	Nd	0.54 ± 0.02	0.44 ± 0.02	Nd	0.61 ± 0.01	0.17 ± 0.23	Nd	0.44 ± 0.02	Nd	Nd	0.39 ± 0.01	Nd
3.3⁣′-Demethyl-heliotropamide	Nd	Nd	0.46 ± 0.12	Nd	Nd	Nd	Nd	Nd	0.35 ± 0.01	Nd	Nd	Nd	Nd
Cannabisin Q	0.60 ± 0.03	Nd	1.56 ± 0.01	0.69 ± 0.02	Nd	0.75 ± 0.07	0.54 ± 0.03	Nd	0.69 ± 0.02	Nd	Nd	1.04 ± 0.06	1.02 ± 0.05
Cannabisin F	0.40 ± 0.02	Nd	0.75 ± 0.02	0.59 ± 0.01	Nd	0.55 ± 0.01	0.42 ± 0.03	Nd	0.59 ± 0.01	0.38 ± 0.01	Nd	0.44 ± 0.02	0.38 ± 0.02
Isocannabisin N	0.56 ± 0.02	0.81 ± 0.02	0.58 ± 0	0.55 ± 0.02	0.62 ± 0.01	0.56 ± 0.00	0.51 ± 0.02	0.76 ± 0.03	0.55 ± 0.02	0.54 ± 0.06	0.86 ± 0.01	0.49 ± 0.03	0.52 ± 0.01
Grossamide	0.44 ± 0.01	Nd	1.1 ± 0.03	0.87 ± 0.02	Nd	0.73 ± 0.00	0.40 ± 0.00	Nd	0.87 ± 0.02	0.34 ± 0.01	Nd	0.27 ± 0.02	0.33 ± 0.01
Cannabisin O	0.97 ± 0.02	0.93 ± 0.08	1.6 ± 0.05	1.13 ± 0.1	0.73 ± 0.15	1.18 ± 0.05	1 ± 0.02	0.57 ± 0.03	0.73 ± 0.02	1.17 ± 0.03	0.60 ± 0	1.14 ± 0.01	1.18 ± 0.03
Unnamed lignanamide	Nd	Nd	0.39 ± 0.01	Nd	Nd	Nd	Nd	Nd	Nd	Nd	Nd	Nd	Nd
Total lignanamides	6.50	1.74	16.18	9.24	1.35	10.36	6.61	1.33	11.74	3.96	1.46	6.65	4.61
Total phenylpropanoids	9.01	1.96	26.15	13.78	1.77	16.31	9.05	1.82	19.08	6.22	1.90	9.39	6.12

Other compounds													
Unknown 1	0.85 ± 0.04	Nd	1.76 ± 0.07	1.34 ± 0.01	Nd	1.18 ± 0.00	0.98 ± 0.02	Nd	1.38 ± 0.06	0.74 ± 0.03	Nd	0.80 ± 0.05	0.56 ± 0.01
Unknown 2	0.48 ± 0.01	0.42 ± 0.02	0.48 ± 0.005	0.47 ± 0.01	0.41 ± 0.01	0.52 ± 0.00	0.47 ± 0	0.46 ± 0.01	0.53 ± 0.01	0.50 ± 0.00	0.46 ± 0.01	0.49 ± 0.03	0.48 ± 0.01
Unknown 3	Nd	Nd	0.57 ± 0.11	Nd	Nd	Nd	Nd	Nd	Nd	Nd	Nd	Nd	Nd
Unknown 4	1.30 ± 0.02	1.26 ± 0.04	1.62 ± 0.02	0.85 ± 0.02	1.19 ± 0.03	1.37 ± 0.02	1.33 ± 0.01	1.28 ± 0	1.41 ± 0.03	1.45 ± 0.03	1.24 ± 0.03	1.52 ± 0.03	1.47 ± 0.07
Total phenolic compounds	19.93 ± 1.09	7.54 ± 0.48	44.07 ± 1.19	24.46 ± 0.2	7.51 ± 0.12	29.46 ± 0.72	19.35 ± 0.45	8.17 ± 0.14	33.52 ± 1.74	16.21 ± 0.59	6.54 ± 0.07	21.75 ± 0.69	16.9 ± 0.44

*Note:* Data, which are the mean ± SD of three independent experiments (*n* = 3), were expressed as milligrams per kilogram oil.

Abbreviations: Nd, not detected; Tr, traces.

^a^Phenylamides and lignanamides are expressed in milligrams caffeoyltyramine equivalent per kilogram of oil.

**Table 6:** Observed and predicted values of the studied responses (oil yield, TPC, total tocopherols, carotenoids, chlorophylls, oxidative stability index (OSI), oil quality indices (peroxide value (PV) and conjugated diene and triene), and color (*L*∗, *a*∗, *b*∗, *C*∗⁣_ab_, and *h*_ab_)) in the oil extracted by microwave-assisted extraction under optimal extraction condition (800 W, 7.5% ethanol, and 13.60 min).

**Parameters**	**Observed values (** **m** **e** **a** **n** ± **SD)**	**Predicted values**	**Confidence intervals at 95%**
Oil yield (%)	30.69 ± 0.04	30.67	[30. 29; 31.05]
Total phenolic content (mg GAE/kg oil)	88.55 ± 0.74	86.11	[62.33; 98.03]
Oxidative stability index (hours)	28.60 ± 0.73	26.67	[24.17; 29.34]
Total tocopherols (mg/kg oil)	510.64 ± 6.34	506.67	[493.02; 520.33]
Total carotenoids (mg/kg oil)	27.31 ± 0.83	26.17	[24.80; 27.54]
Total chlorophylls (mg/kg oil)	99.68 ± 0.73	98.18	[95.47; 100.89]
Color			
*L*∗	33.54 ± 0.63	33.30	[32.56; 34.04]
*a*∗	−4.01 ± 0.03	−4.15	[-3.37; −4.92]
*b*∗	3.17 ± 0.08	2.74	[1.38; 4.10]
*C*∗⁣_ab_	5.11 ± 0.07	4.71	[3.208; 6.206]
*h*_ab_	141.72 ± 0.49	140.55	[138.48; 142.622]
Oil quality indices			
Conjugated diene (*λ*232 nm)	2.24 ± 0.06	2.37	[2.106; 2.639]
Conjugated triene (*λ*270 nm)	0.51 ± 0.01	0.54	[0.466; 0.614]
PV (meq O_2_/kg oil)	7.68 ± 0.34	8.55	[7.41; 9.69]
Fatty acids (%)			
Palmitic acid	10.30 ± 1.24	—	—
Stearic acid	3.09 ± 0.50	—	—
Oleic acid	18.23 ± 1.33	—	—
Linoleic acid	50.23 ± 1.54	—	—
*γ*-Linolenic acid	0.98 ± 0.02	—	—
*α*-Linolenic acid	14.09 ± 0.62	—	—
Arachidic acid	0.62 ± 0.20	—	—
Eicosenoic acid	0.18 ± 0.01	—	—
Behenic acid	0.34 ± 0.08	—	—
Lignoceric acid	0.13 ± 0.09	—	—
SFA	15.10 ± 0.36	—	—
MUFA	18.41 ± 1.34	—	—
PUFA	65.30 ± 0.50	—	—
*n*‐6/*n*‐3 ratio	3.65 ± 0.37	—	—

*Note:*Mean ± SD of three independent experiments (*n* = 3).

## Data Availability

The data that support the findings of this study are available from the corresponding author upon reasonable request.

## Supporting Information

Supporting Information Additional supporting information can be found online in the Supporting Information section. The supporting information includes the following tables: Table S1: Phenolic compounds identified by HPLC-DAD/ESI-MS^2^ with the negative mode [M − H]^−^ in microwave-extracted hemp seed oil. Table S2: Phenolic compounds identified by HPLC-DAD/ESI-MS^2^ with the positive mode [M − H]^+^ in microwave-extracted hemp seed oil.
