# Supplementary material for: Microwave-Assisted Extraction of Hemp Seed Oil: Process Optimization for Enhancing Oil Yield and Bioactive Compound Extractability
Source: Int J Food Sci. 2025 Apr 24;2025:7381308. doi: 10.1155/ijfo/7381308 (PMC12045678; doi:10.1155/ijfo/7381308)
Supplement: Supporting Information — Additional supporting information can be found online in the Supporting Information section. The supporting information includes the following tables: Table S1: Phenolic compounds identified by HPLC-DAD/ESI-MS2 with the negative mode [M − H]− in microwave-extracted hemp seed oil. Table S2: Phenolic compounds identified by HPLC-DAD/ESI-MS2 with the positive mode [M − H]+ in microwave-extracted hemp seed oil. [file 7381308.f1.docx]

**Table S1:** Phenolic compounds identified by HPLC-DAD/ESI-MS^2^ with the negative mode [M−H]^-^ in microwave-extracted hemp seed oil.

| **Phenolic compounds** | **Molecular**  **formula** | **RT**  **(min)** | **λmax (nm)** | **[M − H]^−^ calc. (m/z)** | **[M − H]^−^ found (m/z)** | **Error (ppm)** | **Mass fragments (%intensity)** |
| --- | --- | --- | --- | --- | --- | --- | --- |
| Unknown 1 | –– | 10.35 | 264, 292 | –– | 264.772 | –– | 265(100). 263(64). 247(13) |
| Unknown 2 | –– | 13.94 | 292 | –– | 324.889 | –– | 256(100). 245(85). 241(35). 137(21). 138(14). 237(9) |
| *p*-Hydroxybenzoic acid | C_7_H_6_O_3_ | 14.44 | 288, 378 | 138.032 | 137.0311 | -6.52 | 93(100). 137(89). 94(37) |
| Benzoic acid | C_7_H_6_O_2_ | 18.98 | 280, 220 | 122.037 | 121.0372 | 1.63 | Not fragment |
| *p*-coumaric acid | C_9_H_8_O_3_ | 20.8 | 230, 300, 310 | 164.0478 | 163.0480 | 1.3 | 119 (100). 93 (8) |
| *N-trans*-caffeoyltyramine isomer | C_17_H_16_NO_4_ | 22.54 | 200, 284, 315 | 298.108 | 297.109 | 3.35 | 135(100).178(31). 161(20). 298(19). 284(14) |
| *N-trans*-caffeoyltyramine | C_17_H_16_NO_4_ | 25.38 | 220, 250, 294, 318 | 298.108 | 297.1073 | -2.35 | 135(100). 298(58). 161(26). 178(22). 182(17). 136(11) |
| Unknown 3 | –– | 25.88 | 250, 318, 344 | –– | 207.000 | –– | 192(100). 207(62). 193(6). 205(6). 164(2) |
| Cannabisin A | C_34_H_30_O_8_N_2_ | 27.6 | 256 | 594.2 | 593.2022 | 3.7 | 456(100). 593(42). 430(26). 291(6) |
| Cannabisin B | C_34_H_32_O_8_N_2_ | 27.86 | 254, 284, 314, 334 | 596.216 | 595.218 | 3.35 | 485(100). 269(74). 432(68). 430(59). 322(57). 595(44). 456(11). 241(4). 202(4) |
| *N-trans*-coumaroyltyramine | C_17_H_17_O_3_N | 28.32 | 292, 308 | 283.121 | 282.1208 | -0.42 | 145(100). 282(94). 119(73). 134(46). 162(36). 136(27). 238(8) |
| Cannabisin B Isomer 1 | C_34_H_32_O_8_N_2_ | 29 | 264, 284, 314 | 596.216 | 595.213 | -5.03 | 595(100). 485(41). 544(24). 432(20). 322(18). 269(14) |
| Cannabisin B Isomer 2 | C_34_H_32_O_8_N_2_ | 29.46 | 268, 310 | 596.216 | 595.119 | 0.33 | 485(100). 269(64). 432(56). 456(54). 322(46). 409(34) |
| *N*-feruloyltyramine | C_18_H_19_O_4_N | 29.7 | 256, 288, 318 | 313.131 | 312.1287 | -7.34 | 312(100). 178(96). 135(58). 297(44). 176(11). 148(7). 390(58) |
| Unknown 4 | –– | 29.8 | 322 | –– | 607.000 | –– | 444(100). 382(98). 607(91). 470(24). 592(21). 429(19) |
| Demethylgrossamide | C_35_H_34_N_2_O_8_ | 30.43 | 264, 284, 314, 322 | 610.232 | 609.229 | -4.91 | 293(100). 283(7). 609(5). 446(4) |
| Cannabisin C | C_35_H_34_O_8_N_2_ | 31.72 | 284, 322 | 610.232 | 609.231 | -0.71 | 446(100). 609(83). 283(34). 485(16). 322(15). 571(9). 417(7) |
| 3.3-didemethylgrossamide | C_34_H_32_N_2_O_8_ | 32.81 | 284, 324 | 596.216 | 595.2138 | -3.69 | 432(100)-269(99)-458(36)-595(22)-295(10)-338(7)-250(2) |
| Tri-*p*-coumaroylspermidine | C_34_H_37_N_3_O_6_ | 33.1 | 260 | 583.268 | 582.2722 | 7.2 | 462(100). 582(87). 342(76). 316(10). 436(11). 299(4). 217(2). 533(2) |
| Cannabisin E | C_36_H_38_N_2_O_9_ | 33.39 | 292, 310 | 642.258 | 641.2566 | -2.17 | 623(100). 641(95). 489(55). 431(40). 281(21). 591(20). 312(13).604(11) |
| Cannabisin M | C_34_H_32_N_2_O_8_ | 35.04 | 288, 322 | 596.216 | 595.211 | -8.38 | 298(100). 595(27). 431(24). 430(14). 101(11). 307(10). 467(7). 485(6). 176(1) |
| Unnamed Lignanamide | –– | 35.54 | 278 | –– | 589.266 | –– | 426(100). 589(28). 443(7). 163(6). 261(6). 279(5). 187(3) |
| Cannabisin Q | C_34_H_32_N_2_O_8_ | 36.02 | 284, 308 | 596.216 | 595.2182 | 3.7 | 298(100). 595(45). 296(4). 178(0.5) |
| Cannabisin F | C_36_H_36_N_2_O_8_ | 36.7 | 288, 312 | 624.247 | 623.2433 | -5.92 | 460(100). 623(61). 297(35). 486(29). 352(5) |
| Isocannabisin N | C_35_H_34_N_2_O_8_ | 37.04 | 284, 324 | 610.232 | 609.2336 | 2.62 | 609(100). 312(75). 296(72). 417(16). 723(17). 176(8).561(6) |
| Grossamide | C_36_H_36_N_2_O_8_ | 37.63 | 250, 288, 320 | 624.247 | 623.249 | 3.20 | 623(100). 460(77). 591(47). 297(32). 471(30). 551(23). 432(17). 486(15). 428(11). 282(11) |
| Cannabisin O | C_54_H_53_N_3_O_12_ | 38.09 | 288, 312 | 935.363 | 934.361 | -1.17 | Not fragment |
| Sinapic acid | C_11_H_12_O_5_ | 48.81 | 276 | 224.069 | 223.0677 | -5.49 | 225(100). 223(34). 195(36). 125(35). 179(24). 221(20). 163(18). 206(16). 164(12). 155(17) |

RT, retention time; λmax, maximum absorbance peak

**Table S2:** Phenolic compounds identified by HPLC-DAD/ESI-MS^2^ with the positive mode [M−H]^+^ in microwave-extracted hemp seed oil.

| **Phenolic compounds** | **Molecular**  **formula** | **RT**  **(min)** | **λmax (nm)** | **[M − H]^+^**  **calc. (m/z)** | **[M − H]^+^ Found (m/z)** | **Error (ppm)** | **Mass fragments (%intensity)** |
| --- | --- | --- | --- | --- | --- | --- | --- |
| Benzoic acid | C_7_H_6_O_2_ | 18.98 | 280, 220 | 122.037 | 123.0372 | 4.09 | 123(100). 95(74). 122(20). 79(17). 97(15). 107(14). 96(12). 20(7) |
| *N-trans-*caffeoyltyramine isomer | C_17_H_16_NO_4_ | 22.54 | 200, 284, 315 | 298.108 | 299.1084 | 1.34 | 163(100). 300(27). 138(14). 121(10). 145(5). 117(2). 283(2) |
| *N-trans*-caffeoyltyramine | C_17_H_16_NO_4_ | 25.38 | 220, 250, 294, 318 | 298.108 | 299.1075 | -1.67 | 163(100). 300(55). 138(20). 121(10). 164(12). 145(4) |
| Unknown 3 | –– | 25.88 | 250, 318, 344 | –– | 208.8609 | –– | 177(100). 209(83). 191(41). 149(15). 121(13). 131(7). 145(10). 153(5). 181(6) |
| Cannabisin A | C_34_H_30_O_8_N_2_ | 27.6 | 256 | 594.2 | 594.196 | -6.73 | 485(100). 595(41). 593(6). 336(4) |
| Cannabisin B | C_34_H_32_O_8_N_2_ | 27.86 | 254, 284, 314, 334 | 596.216 | 597.2125 | -5.87 | 460(100). 432(22). 597(14). 350(10). 295(7). 844(7). 187(4) |
| *N-trans*-coumaroyltyramine | C_17_H_17_O_3_N | 28.32 | 292, 308 | 283.121 | 284.120 | -3.53 | 147(100). 284(65). 148(17). 119(2) |
| Cannabisin B isomer 1 | C_34_H_32_O_8_N_2_ | 29 | 264, 284, 314 | 596.216 | 597.2122 | -6.37 | 460(100). 434(55). 432(25). 597(20). 350(14). 258(13). 295(10) |
| Cannabisin B Isomer 2 | C_34_H_32_O_8_N_2_ | 29.46 | 268, 310 | 596.216 | 597.2137 | -3.85 | 460(100). 432(32). 597(25). 295(13). 350(7). 418(5). 279(5) |
| *N*-feruloyltyramine | C_18_H_19_O_4_N | 29.7 | 256, 288, 318 | 313.131 | 314.130 | -3.19 | 177(100). 178(75). 314(18). 145(15). 298(3). 117(3) |
| Demethylgrossamide | C_35_H_34_N_2_O_8_ | 30.43 | 264, 284, 314, 322 | 610.232 | 611.2439 | 3.11 | 474(100). 612(65). 446(22). 350(11). 309(4). 564(2). 187(1). 591(1) |
| Cannabisin C | C_35_H_34_O_8_N_2_ | 31.72 | 284, 322 | 610.232 | 611.2319 | -0.16 | 611(100). 474(57). 311(23). 446(16). 448(9). 350(6). 309(6). 432(5). 265(4) |
| Tri-*p*-coumaroylspermidine | C_34_H_37_N_3_O_6_ | 33.1 | 260 | 583.268 | 584.2646 | -5.82 | 420(100). 438(97). 584(34). 585(11). 204(6). 217(4). |
| Cannabisin Q | C_34_H_32_N_2_O_8_ | 36.02 | 284, 308 | 596.216 | 597.2032 | -21.46 | 297(100). 597(31). 460(24). 434(21). 187(21). 279(11). 233(5). 251(5) |
| Cannabisin F | C_36_H_36_N_2_O_8_ | 36.7 | 288, 312 | 624.247 | 625.243 | -6.40 | 462(100). 625(70). 325(44). 307(12). 351(8). 293(5). 201(2) |
| Isocannabisin N | C_35_H_34_N_2_O_8_ | 37.04 | 284, 324 | 610.232 | 611.2318 | -0.32 | 311(100). 448(50). 187(38). 611(30). 350(18). 470(15).591(15) |
| Grossamide | C_36_H_36_N_2_O_8_ | 37.63 | 250, 288, 320 | 624.247 | 625.2466 | -0.64 | 625(100). 462(98). 325(85). 351(67). 488(54). 307(46). 293(24). 201(10) |
| Cannabisin O | C_54_H_53_N_3_O_12_ | 38.09 | 284 | 935.363 | 936.359 | -4.27 | 799(100). 538(36). 634(30). 771(21). 512(15). 936(12). 675(8). 388(6) |

RT, retention time; λmax, maximum absorbance peak
